# Supplementary material for: Frequent introductions and climate suitability drive increasing dengue risk in Florida
Source: medRxiv. 2026 May 5:2026.05.01.26352185. Preprint. [Version 2] doi: 10.64898/2026.05.01.26352185 (PMC13174716; doi:10.64898/2026.05.01.26352185)
Supplement: Supplement 1 [file NIHPP2026.05.01.26352185v2-supplement-1.pdf]

medRxiv preprint doi: https://doi.org/10.64898/2026.05.01.26352185; this version posted May 5, 2026. The copyright holder for this preprint (which was not certified by peer review) is the author/funder, who has granted medRxiv a license to display the preprint in perpetuity.
It is made available under a CC-BY 4.0 International license .

# Supplemental Figures & Tables

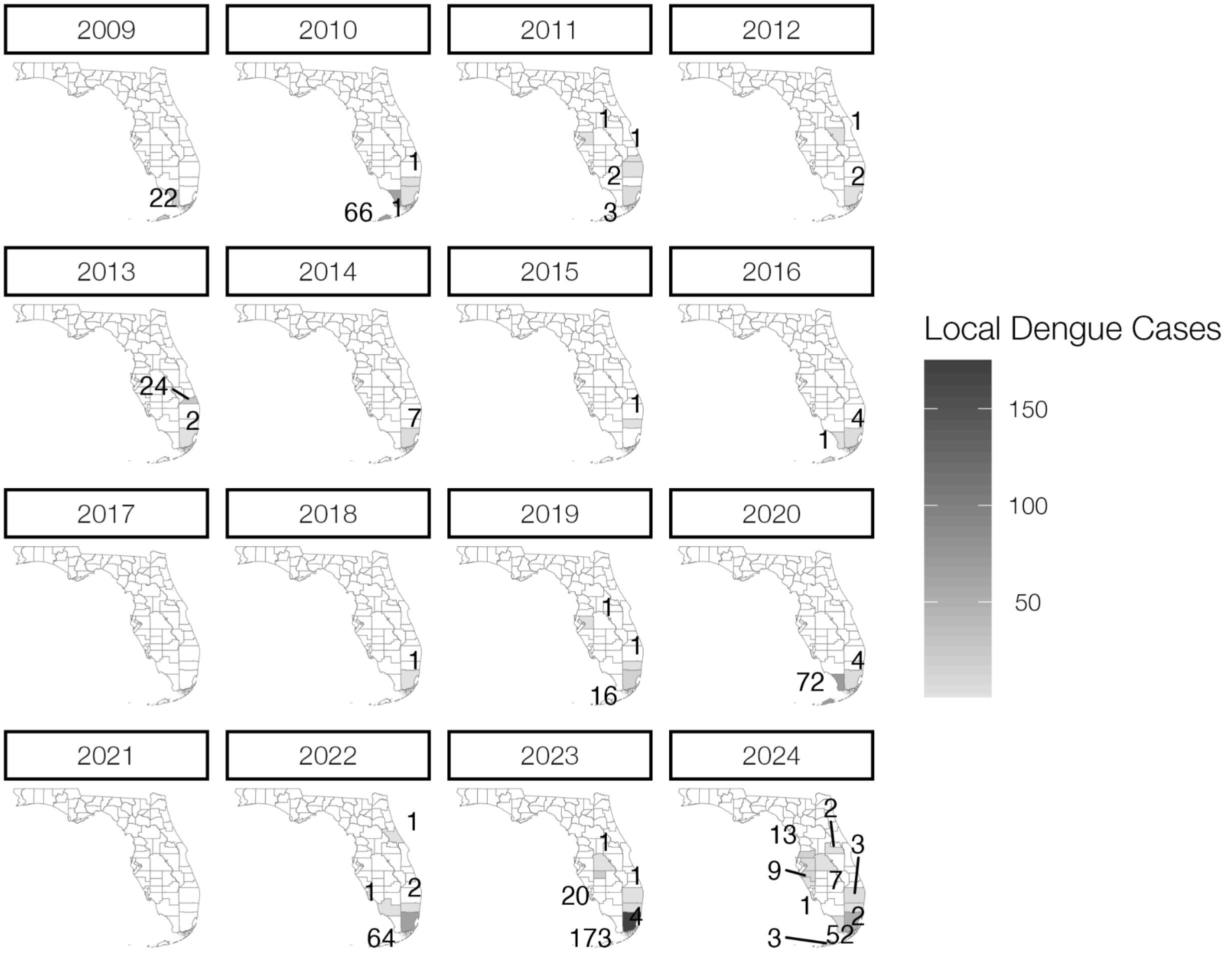


**Supplemental Figure 1.** Total number of local dengue cases per county reported in Florida from 2009 to 2024. There were no local cases reported in 2017 or 2021.

medRxiv preprint doi: https://doi.org/10.64898/2026.05.01.26352185; this version posted May 5, 2026. The copyright holder for this preprint (which was not certified by peer review) is the author/funder, who has granted medRxiv a license to display the preprint in perpetuity.
It is made available under a CC-BY 4.0 International license .

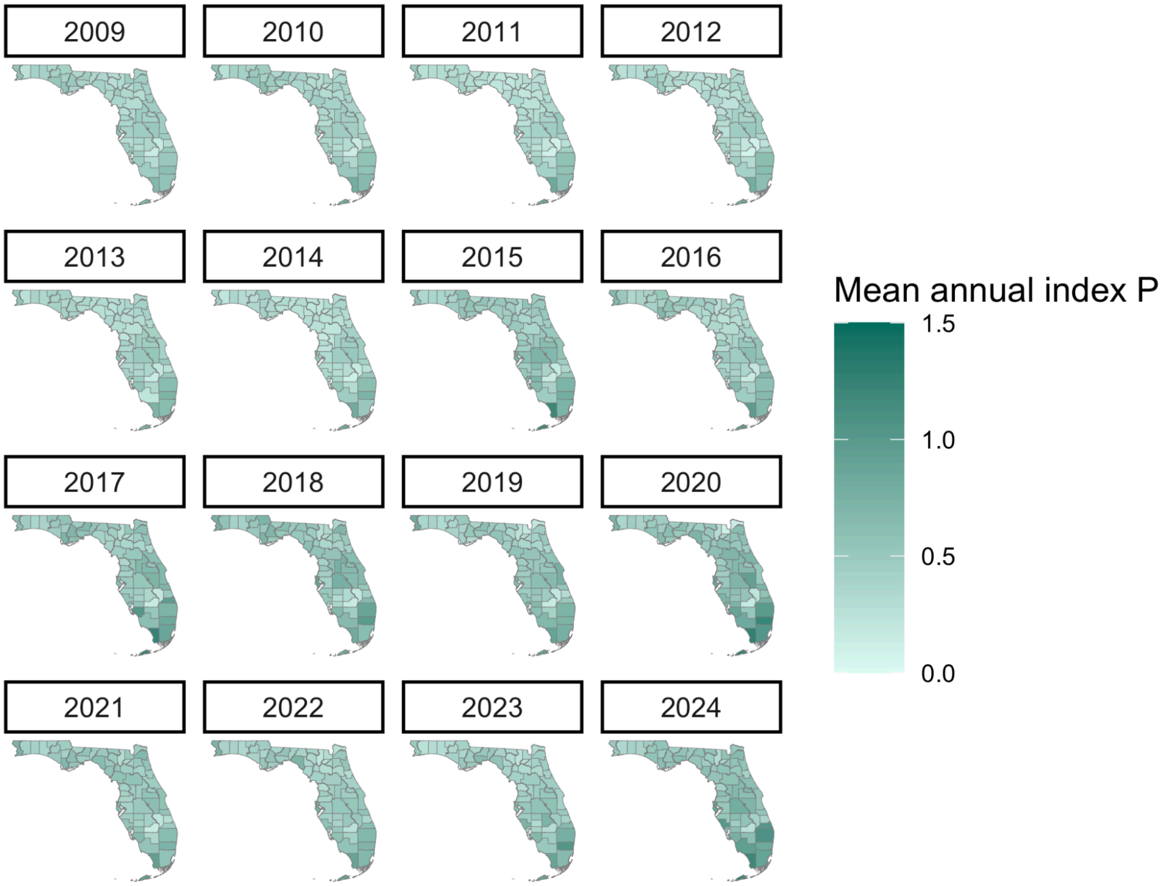


**Supplemental Figure 2.** Yearly estimated mean index P for DENV transmitted by *Aedes aegypti* mosquitoes per county in Florida from 2009 to 2024.

medRxiv preprint doi: https://doi.org/10.64898/2026.05.01.26352185; this version posted May 5, 2026. The copyright holder for this preprint (which was not certified by peer review) is the author/funder, who has granted medRxiv a license to display the preprint in perpetuity.
It is made available under a CC-BY 4.0 International license .

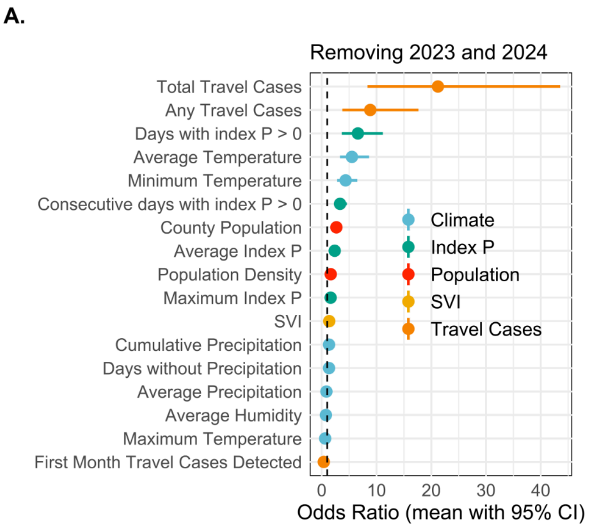


**Supplemental Figure 3.** Sensitivity analysis to understand the marginal linear fixed effects of annual local dengue case occurrence by county by various covariates if (A) the 2023-2024 are removed from the analysis or (B) Miami-Dade County were removed from the analysis. The circles indicate the predicted mean and the lines indicate the 95% confidence interval. Covariates are colored by category.

medRxiv preprint doi: https://doi.org/10.64898/2026.05.01.26352185; this version posted May 5, 2026. The copyright holder for this preprint (which was not certified by peer review) is the author/funder, who has granted medRxiv a license to display the preprint in perpetuity.
It is made available under a CC-BY 4.0 International license .

**Table S1.** Genomic dataset including all travel-associated (n=294) and locally acquired (n=133) dengue cases reported by the FDOH from 2009-2024, and a global background dataset (n=5,153) filtered for major lineages matching those of locally acquired dengue cases. Sequence counts are broken down by sample collection location and serotype. Sample collection locations are grouped into traits for phylogeographic inference (Region(DTA)).

| Location | Region (DTA) | DENV-1 | DENV-2 | DENV-3 | DENV-4 |
|---|---|---|---|---|---|
| Florida (USA) | Alachua | 0 | 1 | 0 | 0 |
| Florida (USA) | Hardee | 1 | 0 | 0 | 0 |
| Florida (USA) | Hillsborough | 0 | 0 | 1 | 0 |
| Florida (USA) | Manatee | 0 | 0 | 0 | 1 |
| Florida (USA) | Martin | 1 | 0 | 0 | 0 |
| Florida (USA) | Miami-Dade | 1 | 14 | 95 | 2 |
| Florida (USA) | Monroe | 12 | 0 | 0 | 0 |
| Florida (USA) | Orange | 0 | 0 | 1 | 0 |
| Florida (USA) | Palm-Beach | 0 | 0 | 1 | 0 |
| Florida (USA) | Pasco | 0 | 0 | 1 | 0 |
| Florida (USA) | Polk | 0 | 0 | 1 | 0 |
| Barbados | Caribbean | 0 | 1 | 0 | 0 |
| Cuba | Caribbean | 23 | 98 | 293 | 48 |
| Dominica | Caribbean | 0 | 0 | 0 | 2 |
| Dominican-Republic | Caribbean | 8 | 49 | 68 | 2 |
| Guadeloupe | Caribbean | 4 | 50 | 2 | 0 |
| Haiti | Caribbean | 6 | 3 | 2 | 5 |
| Jamaica | Caribbean | 2 | 9 | 10 | 1 |
| Martinique | Caribbean | 0 | 55 | 7 | 0 |
| Puerto-Rico | Caribbean | 26 | 11 | 13 | 29 |
| Saint-Barthelemy | Caribbean | 1 | 4 | 0 | 0 |
| Saint-Croix | Caribbean | 0 | 1 | 0 | 0 |
| Saint-Kitts-and-Nevis | Caribbean | 0 | 1 | 0 | 0 |
| Saint-Lucia | Caribbean | 0 | 0 | 2 | 0 |
| Saint-Martin | Caribbean | 3 | 1 | 0 | 0 |
| Trinidad-and-Tobago | Caribbean | 0 | 1 | 7 | 0 |
| US-Virgin-Islands | Caribbean | 0 | 1 | 0 | 0 |
| Belize | Central-America | 0 | 1 | 0 | 0 |
| Costa-Rica | Central-America | 2 | 1 | 5 | 0 |
| El-Salvador | Central-America | 0 | 0 | 2 | 2 |
| Guatemala | Central-America | 0 | 3 | 8 | 0 |
| Honduras | Central-America | 1 | 5 | 13 | 0 |
| Nicaragua | Central-America | 63 | 289 | 0 | 6 |
| Panama | Central-America | 0 | 1 | 1 | 2 |
| Angola | Global | 0 | 0 | 1 | 0 |
| Australia | Global | 0 | 2 | 2 | 0 |
| Bangladesh | Global | 2 | 8 | 36 | 0 |
| Benin | Global | 1 | 0 | 0 | 0 |
| Bhutan | Global | 0 | 0 | 44 | 0 |
| Brunei | Global | 1 | 2 | 0 | 0 |
| Burkina-Faso | Global | 6 | 0 | 8 | 0 |

medRxiv preprint doi: https://doi.org/10.64898/2026.05.01.26352185; this version posted May 5, 2026. The copyright holder for this preprint (which was not certified by peer review) is the author/funder, who has granted medRxiv a license to display the preprint in perpetuity.
It is made available under a CC-BY 4.0 International license .

| | | | | | |
|---|---|---|---|---|---|
| Cambodia | Global | 1 | 114 | 1 | 0 |
| Cameroon | Global | 0 | 1 | 0 | 0 |
| China | Global | 109 | 277 | 75 | 0 |
| Djibouti | Global | 1 | 0 | 0 | 0 |
| Egypt | Global | 0 | 1 | 0 | 0 |
| Eritrea | Global | 1 | 0 | 0 | 0 |
| Ethiopia | Global | 0 | 0 | 2 | 0 |
| France | Global | 1 | 4 | 0 | 0 |
| Gabon | Global | 0 | 0 | 5 | 0 |
| Ghana | Global | 1 | 0 | 0 | 0 |
| Guam | Global | 0 | 1 | 0 | 0 |
| India | Global | 73 | 26 | 59 | 1 |
| Indonesia | Global | 0 | 10 | 18 | 4 |
| Israel | Global | 2 | 0 | 2 | 0 |
| Italy | Global | 7 | 0 | 3 | 0 |
| Ivory-Coast | Global | 3 | 0 | 0 | 0 |
| Kenya | Global | 5 | 0 | 5 | 0 |
| Kiribati | Global | 0 | 0 | 0 | 1 |
| Malaysia | Global | 2 | 43 | 4 | 0 |
| Maldives | Global | 0 | 1 | 4 | 0 |
| Mauritania | Global | 1 | 0 | 0 | 0 |
| Mayotte | Global | 31 | 0 | 0 | 0 |
| Myanmar | Global | 0 | 0 | 39 | 0 |
| Nepal | Global | 4 | 23 | 2 | 0 |
| Niger | Global | 0 | 0 | 1 | 0 |
| Nigeria | Global | 3 | 0 | 0 | 0 |
| Niue | Global | 0 | 0 | 0 | 1 |
| Pakistan | Global | 23 | 14 | 6 | 0 |
| Papua-New-Guinea | Global | 0 | 0 | 5 | 0 |
| Philippines | Global | 0 | 2 | 0 | 0 |
| Reunion | Global | 43 | 5 | 17 | 0 |
| Saudi-Arabia | Global | 0 | 0 | 1 | 0 |
| Senegal | Global | 3 | 0 | 12 | 1 |
| Seychelles | Global | 1 | 0 | 0 | 0 |
| Singapore | Global | 102 | 124 | 14 | 2 |
| South-Korea | Global | 0 | 1 | 0 | 0 |
| Sri-Lanka | Global | 39 | 156 | 58 | 0 |
| Taiwan | Global | 0 | 1 | 0 | 0 |
| Tanzania | Global | 2 | 0 | 0 | 0 |
| Thailand | Global | 0 | 13 | 60 | 0 |
| Timor-Leste | Global | 0 | 0 | 4 | 0 |
| UAE | Global | 0 | 1 | 0 | 0 |
| Vietnam | Global | 0 | 50 | 0 | 0 |
| Arizona (USA) | North-America | 0 | 0 | 3 | 0 |
| California (USA) | North-America | 1 | 5 | 28 | 0 |
| Mexico | North-America | 127 | 34 | 12 | 4 |
| Texas (USA) | North-America | 1 | 5 | 0 | 0 |
| Argentina | South-America | 20 | 1 | 0 | 0 |

medRxiv preprint doi: https://doi.org/10.64898/2026.05.01.26352185; this version posted May 5, 2026. The copyright holder for this preprint (which was not certified by peer review) is the author/funder, who has granted medRxiv a license to display the preprint in perpetuity.
It is made available under a CC-BY 4.0 International license .

| | | | | | |
|---|---|---|---|---|---|
| Bolivia | South-America | 3 | 1 | 0 | 1 |
| Brazil | South-America | 753 | 602 | 9 | 87 |
| Colombia | South-America | 19 | 121 | 1 | 12 |
| Ecuador | South-America | 16 | 33 | 0 | 2 |
| French-Guiana | South-America | 0 | 12 | 6 | 0 |
| Guyana | South-America | 0 | 0 | 2 | 0 |
| Paraguay | South-America | 40 | 37 | 0 | 98 |
| Peru | South-America | 51 | 59 | 10 | 0 |
| Suriname | South-America | 0 | 2 | 0 | 0 |
| Venezuela | South-America | 35 | 46 | 1 | 47 |

medRxiv preprint doi: https://doi.org/10.64898/2026.05.01.26352185; this version posted May 5, 2026. The copyright holder for this preprint (which was not certified by peer review) is the author/funder, who has granted medRxiv a license to display the preprint in perpetuity.
It is made available under a CC-BY 4.0 International license .

**Table S2. Genbank accession numbers, collection dates & regions, and lineage information for sequences sampled from either local (Local=1, n=133) or travel-associated (Travel=1, n=294) dengue cases in Florida.**

| GenBank Accession | Year | Month | Day | Local | Travel | Location | Region (DTA) | Serotype | Lineage |
|---|---|---|---|---|---|---|---|---|---|
| OQ821370 | 2010 | 6 | 24 | 1 | 0 | Florida (USA) | Monroe | 1 | 1V_C |
| OQ821371 | 2010 | 6 | 30 | 1 | 0 | Florida (USA) | Monroe | 1 | 1V_C |
| OQ821353 | 2010 | 7 | 28 | 0 | 1 | Dominican-Republic | Caribbean (Travel) | 1 | 1V_E |
| OQ821494 | 2010 | 7 | 30 | 0 | 1 | Puerto-Rico | Caribbean (Travel) | 2 | 2III_B |
| OQ821632 | 2010 | 8 | 5 | 0 | 1 | Puerto-Rico | Caribbean (Travel) | 4 | 4II_B |
| OQ821372 | 2010 | 8 | 6 | 1 | 0 | Florida (USA) | Monroe | 1 | 1V_C |
| OQ821373 | 2010 | 8 | 11 | 1 | 0 | Florida (USA) | Monroe | 1 | 1V_C |
| OQ821374 | 2010 | 8 | 27 | 1 | 0 | Florida (USA) | Monroe | 1 | 1V_C |
| OQ821375 | 2010 | 10 | 11 | 1 | 0 | Florida (USA) | Monroe | 1 | 1V_C |
| JQ675358 | 2010 | 10 |  | 1 | 0 | Florida (USA) | Monroe | 1 | 1V_C |
| OQ821376 | 2010 | 11 | 20 | 1 | 0 | Florida (USA) | Monroe | 1 | 1V_C |
| OQ821628 | 2012 | 8 | 20 | 0 | 1 | Cuba | Caribbean (Travel) | 4 | 4II_B.1 |
| OQ821364 | 2012 | 9 | 2 | 0 | 1 | Jamaica | Caribbean (Travel) | 1 | 1V_E |
| OQ821629 | 2012 | 10 | 3 | 0 | 1 | Cuba | Caribbean (Travel) | 4 | 4II_B.1 |
| OM833059 | 2013 | 7 | 24 | 1 | 0 | Florida (USA) | Monroe | 1 | 1V_E.3 |
| OQ821650 | 2013 | 8 | 21 | 0 | 1 | Dominica | Caribbean (Travel) | 4 | 4II_A.1 |
| OQ821377 | 2013 | 8 | 29 | 1 | 0 | Florida (USA) | Martin | 1 | 1V_E.2 |
| OQ821633 | 2013 | 9 | 6 | 0 | 1 | Haiti | Caribbean (Travel) | 4 | 4II_B |
| OQ821354 | 2013 | 9 | 21 | 0 | 1 | Dominican-Republic | Caribbean (Travel) | 1 | 1V_E.2 |
| OQ821383 | 2013 | 12 | 1 | 0 | 1 | Cuba | Caribbean (Travel) | 2 | 2III_D.1.2 |
| OQ821626 | 2013 | 12 | 6 | 0 | 1 | Cuba | Caribbean (Travel) | 4 | 4II_B.1 |
| OQ821472 | 2014 | 1 | 12 | 0 | 1 | Dominican-Republic | Caribbean (Travel) | 2 | 2III_C.1 |
| OQ821630 | 2014 | 3 | 13 | 0 | 1 | Bolivia | South-America (Travel) | 4 | 4II_B.1.1 |
| OQ821612 | 2014 | 7 | 2 | 1 | 0 | Florida (USA) | Miami-Dade | 3 | 3I_A |
| OQ821627 | 2014 | 8 | 6 | 0 | 1 | Cuba | Caribbean (Travel) | 4 | 4II_B.1 |
| OQ821476 | 2014 | 10 | 14 | 0 | 1 | Honduras | Central-America (Travel) | 2 | 2III_D.1.2 |
| OQ821327 | 2015 | 5 | 19 | 0 | 1 | Brazil | South-America (Travel) | 1 | 1V_F |
| OQ821500 | 2015 | 8 | 31 | 0 | 1 | Venezuela | South-America (Travel) | 2 | 2III_D.2 |
| OQ821475 | 2015 | 10 | 28 | 0 | 1 | Haiti | Caribbean (Travel) | 2 | 2III_C.1.2 |
| OQ821503 | 2016 | 1 | 13 | 0 | 1 | Cuba | Caribbean (Travel) | 3 | 3III_C.1 |
| OQ821501 | 2016 | 1 | 23 | 0 | 1 | Venezuela | South-America (Travel) | 2 | 2III_D.2 |
| OQ821625 | 2016 | 2 | 5 | 0 | 1 | Cuba | Caribbean (Travel) | 4 | 4II_B.1 |
| OQ821328 | 2016 | 2 | 8 | 0 | 1 | Brazil | South-America (Travel) | 1 | 1V_A |
| OQ821332 | 2016 | 3 | 15 | 0 | 1 | Costa-Rica | Central-America (Travel) | 1 | 1V_C |
| OQ821649 | 2016 | 3 | 21 | 0 | 1 | Jamaica | Caribbean (Travel) | 4 | 4II_A.1 |
| KX702404 | 2016 | 5 | 22 | 1 | 0 | Florida (USA) | Alachua | 2 | 2III_C |
| OQ821601 | 2016 | 6 | 11 | 0 | 1 | Jamaica | Caribbean (Travel) | 3 | 3III_B.3.1 |
| OQ821331 | 2016 | 7 | 12 | 0 | 1 | Colombia | South-America (Travel) | 1 | 1V_D.1 |
| OQ821602 | 2016 | 7 | 27 | 0 | 1 | Jamaica | Caribbean (Travel) | 3 | 3III_B.3.1 |

medRxiv preprint doi: https://doi.org/10.64898/2026.05.01.26352185; this version posted May 5, 2026. The copyright holder for this preprint (which was not certified by peer review) is the author/funder, who has granted medRxiv a license to display the preprint in perpetuity.
It is made available under a CC-BY 4.0 International license .

| | | | | | | | | | |
|---|---|---|---|---|---|---|---|---|---|
| OQ821381 | 2016 | 7 | 28 | 0 | 1 | Colombia | South-America (Travel) | 2 | 2III_D.3 |
| OQ821382 | 2016 | 8 | 1 | 0 | 1 | Costa-Rica | Central-America (Travel) | 2 | 2III_D.1.1 |
| OQ821603 | 2016 | 9 | 12 | 0 | 1 | Jamaica | Caribbean (Travel) | 3 | 3III_B.3.1 |
| MN192436 | 2016 | | | 1 | 0 | Florida (USA) | Manatee | 4 | 4II_B |
| OQ821474 | 2017 | 4 | 22 | 0 | 1 | Guatemala | Central-America (Travel) | 2 | 2III_D.1.2 |
| OQ821384 | 2017 | 6 | 29 | 0 | 1 | Cuba | Caribbean (Travel) | 2 | 2III_C.2 |
| OQ821480 | 2017 | 9 | 8 | 0 | 1 | India | South-Asia (Travel) | 2 | 2II_A.2.1 |
| OQ821360 | 2018 | 7 | 5 | 0 | 1 | Haiti | Caribbean (Travel) | 1 | 1V_E.2 |
| OQ821385 | 2018 | 11 | 4 | 0 | 1 | Cuba | Caribbean (Travel) | 2 | 2III_C.2 |
| OQ821386 | 2018 | 11 | 20 | 0 | 1 | Cuba | Caribbean (Travel) | 2 | 2III_C.2 |
| OQ821387 | 2018 | 12 | 5 | 0 | 1 | Cuba | Caribbean (Travel) | 2 | 2III_C.2 |
| OQ821388 | 2019 | 1 | 6 | 0 | 1 | Cuba | Caribbean (Travel) | 2 | 2III_C.2 |
| OQ821379 | 2019 | 1 | 7 | 0 | 1 | Venezuela | South-America (Travel) | 1 | 1V_D.1 |
| OQ821389 | 2019 | 1 | 13 | 0 | 1 | Cuba | Caribbean (Travel) | 2 | 2III_C.2 |
| OQ821604 | 2019 | 1 | 15 | 0 | 1 | Jamaica | Caribbean (Travel) | 3 | 3III_B.3.1 |
| OQ821605 | 2019 | 2 | 1 | 0 | 1 | Jamaica | Caribbean (Travel) | 3 | 3III_B.3.1 |
| OQ821390 | 2019 | 3 | 20 | 0 | 1 | Cuba | Caribbean (Travel) | 2 | 2III_C.2 |
| OQ821391 | 2019 | 3 | 20 | 0 | 1 | Cuba | Caribbean (Travel) | 2 | 2III_C.2 |
| OQ821392 | 2019 | 6 | 3 | 0 | 1 | Cuba | Caribbean (Travel) | 2 | 2III_D.1.1 |
| OQ821486 | 2019 | 6 | 6 | 0 | 1 | Nicaragua | Central-America (Travel) | 2 | 2III_D.1.2 |
| OQ821393 | 2019 | 6 | 7 | 0 | 1 | Cuba | Caribbean (Travel) | 2 | 2III_C.2 |
| OQ821394 | 2019 | 6 | 29 | 0 | 1 | Cuba | Caribbean (Travel) | 2 | 2III_C.2 |
| OQ821355 | 2019 | 7 | 4 | 0 | 1 | Dominican-Republic | Caribbean (Travel) | 1 | 1V_E.2 |
| OQ821395 | 2019 | 7 | 10 | 0 | 1 | Cuba | Caribbean (Travel) | 2 | 2III_C.2 |
| OQ821396 | 2019 | 7 | 10 | 0 | 1 | Cuba | Caribbean (Travel) | 2 | 2III_C.2 |
| OQ821397 | 2019 | 7 | 11 | 0 | 1 | Cuba | Caribbean (Travel) | 2 | 2III_C.2 |
| OQ821496 | 2019 | 7 | 11 | 1 | 0 | Florida (USA) | Miami-Dade | 2 | 2III_D.1.1 |
| OQ821477 | 2019 | 7 | 12 | 0 | 1 | Honduras | Central-America (Travel) | 2 | 2III_D.1.2 |
| OQ821487 | 2019 | 7 | 14 | 0 | 1 | Nicaragua | Central-America (Travel) | 2 | 2III_D.1.2 |
| OQ821398 | 2019 | 7 | 15 | 0 | 1 | Cuba | Caribbean (Travel) | 2 | 2III_C.2 |
| OQ821606 | 2019 | 7 | 15 | 0 | 1 | Jamaica | Caribbean (Travel) | 3 | 3III_B.3.1 |
| OQ821330 | 2019 | 7 | 17 | 0 | 1 | Cambodia | Southeast-Asia (Travel) | 1 | 1I_E.1 |
| OQ821399 | 2019 | 7 | 21 | 0 | 1 | Cuba | Caribbean (Travel) | 2 | 2III_C.2 |
| OQ821400 | 2019 | 7 | 22 | 0 | 1 | Cuba | Caribbean (Travel) | 2 | 2III_C.2 |
| OQ821401 | 2019 | 7 | 24 | 0 | 1 | Cuba | Caribbean (Travel) | 2 | 2III_C.2 |
| OQ821333 | 2019 | 7 | 26 | 0 | 1 | Cuba | Caribbean (Travel) | 1 | 1V_D.1.1 |
| OQ821402 | 2019 | 7 | 27 | 0 | 1 | Cuba | Caribbean (Travel) | 2 | 2III_C.2 |
| OQ821403 | 2019 | 7 | 27 | 0 | 1 | Cuba | Caribbean (Travel) | 2 | 2III_C.2 |
| OQ821404 | 2019 | 7 | 28 | 0 | 1 | Cuba | Caribbean (Travel) | 2 | 2III_C.2 |
| OQ821493 | 2019 | 7 | 31 | 0 | 1 | Philippines | Southeast-Asia (Travel) | 2 | 2II_C.1 |
| OQ821405 | 2019 | 8 | 1 | 0 | 1 | Cuba | Caribbean (Travel) | 2 | 2III_C.2 |
| OQ821478 | 2019 | 8 | 2 | 0 | 1 | Honduras | Central-America (Travel) | 2 | 2III_D.1.2 |
| OQ821406 | 2019 | 8 | 3 | 0 | 1 | Cuba | Caribbean (Travel) | 2 | 2III_C.2 |

medRxiv preprint doi: https://doi.org/10.64898/2026.05.01.26352185; this version posted May 5, 2026. The copyright holder for this preprint (which was not certified by peer review) is the author/funder, who has granted medRxiv a license to display the preprint in perpetuity.
It is made available under a CC-BY 4.0 International license .

| | | | | | | | | | |
|---|---|---|---|---|---|---|---|---|---|
| OQ821407 | 2019 | 8 | 3 | 0 | 1 | Cuba | Caribbean (Travel) | 2 | 2III_D.1.1 |
| OQ821408 | 2019 | 8 | 6 | 0 | 1 | Cuba | Caribbean (Travel) | 2 | 2III_C.2 |
| OQ821356 | 2019 | 8 | 12 | 0 | 1 | Dominican-Republic | Caribbean (Travel) | 1 | 1V_E.2 |
| OQ821361 | 2019 | 8 | 12 | 0 | 1 | Haiti | Caribbean (Travel) | 1 | 1V_E.2 |
| OQ821607 | 2019 | 8 | 12 | 0 | 1 | Jamaica | Caribbean (Travel) | 3 | 3III_B.3.1 |
| OQ821488 | 2019 | 8 | 12 | 0 | 1 | Nicaragua | Central-America (Travel) | 2 | 2III_D.1.2 |
| OQ821362 | 2019 | 8 | 14 | 0 | 1 | Haiti | Caribbean (Travel) | 1 | 1V_E.2 |
| OQ821334 | 2019 | 8 | 18 | 0 | 1 | Cuba | Caribbean (Travel) | 1 | 1V_D.1.1 |
| OQ821483 | 2019 | 8 | 19 | 0 | 1 | Mexico | North-America (Travel) | 2 | 2III_D.1.2 |
| OQ821409 | 2019 | 8 | 22 | 0 | 1 | Cuba | Caribbean (Travel) | 2 | 2III_C.2 |
| OQ821363 | 2019 | 8 | 23 | 0 | 1 | Honduras | Central-America (Travel) | 1 | 1V_C |
| OQ821410 | 2019 | 8 | 25 | 0 | 1 | Cuba | Caribbean (Travel) | 2 | 2III_C.2 |
| OQ821411 | 2019 | 8 | 26 | 0 | 1 | Cuba | Caribbean (Travel) | 2 | 2III_C.2 |
| OQ821412 | 2019 | 8 | 26 | 0 | 1 | Cuba | Caribbean (Travel) | 2 | 2III_C.2 |
| OQ821479 | 2019 | 8 | 27 | 0 | 1 | Honduras | Central-America (Travel) | 2 | 2III_D.1.2 |
| OQ821489 | 2019 | 8 | 27 | 0 | 1 | Nicaragua | Central-America (Travel) | 2 | 2III_D.1.2 |
| OQ821335 | 2019 | 8 | 28 | 0 | 1 | Cuba | Caribbean (Travel) | 1 | 1V_E.2 |
| OQ821413 | 2019 | 8 | 28 | 0 | 1 | Cuba | Caribbean (Travel) | 2 | 2III_C.2 |
| OQ821497 | 2019 | 8 | 29 | 1 | 0 | Florida (USA) | Miami-Dade | 2 | 2III_D.1.2 |
| OQ821414 | 2019 | 8 | 30 | 0 | 1 | Cuba | Caribbean (Travel) | 2 | 2III_C.2 |
| OQ821415 | 2019 | 8 | 30 | 0 | 1 | Cuba | Caribbean (Travel) | 2 | 2III_C.2 |
| OQ821416 | 2019 | 9 | 2 | 0 | 1 | Cuba | Caribbean (Travel) | 2 | 2III_C.2 |
| OQ821417 | 2019 | 9 | 4 | 0 | 1 | Cuba | Caribbean (Travel) | 2 | 2III_C.2 |
| OQ821490 | 2019 | 9 | 5 | 0 | 1 | Nicaragua | Central-America (Travel) | 2 | 2III_D.1.2 |
| OQ821418 | 2019 | 9 | 7 | 0 | 1 | Cuba | Caribbean (Travel) | 2 | 2III_C.2 |
| OQ821419 | 2019 | 9 | 8 | 0 | 1 | Cuba | Caribbean (Travel) | 2 | 2III_D.1.1 |
| OQ821420 | 2019 | 9 | 8 | 0 | 1 | Cuba | Caribbean (Travel) | 2 | 2III_D.1.1 |
| OQ821357 | 2019 | 9 | 10 | 0 | 1 | Dominican-Republic | Caribbean (Travel) | 1 | 1V_E.2 |
| OQ821421 | 2019 | 9 | 13 | 0 | 1 | Cuba | Caribbean (Travel) | 2 | 2III_C.2 |
| OQ821422 | 2019 | 9 | 13 | 0 | 1 | Cuba | Caribbean (Travel) | 2 | 2III_C.2 |
| OQ821498 | 2019 | 9 | 14 | 1 | 0 | Florida (USA) | Miami-Dade | 2 | 2III_D.1.2 |
| OQ821424 | 2019 | 9 | 16 | 0 | 1 | Cuba | Caribbean (Travel) | 2 | 2III_C.2 |
| OQ821425 | 2019 | 9 | 18 | 0 | 1 | Cuba | Caribbean (Travel) | 2 | 2III_C.2 |
| OQ821426 | 2019 | 9 | 18 | 0 | 1 | Cuba | Caribbean (Travel) | 2 | 2III_C.2 |
| OQ821428 | 2019 | 9 | 18 | 0 | 1 | Cuba | Caribbean (Travel) | 2 | 2III_D.1.1 |
| OQ821429 | 2019 | 9 | 20 | 0 | 1 | Cuba | Caribbean (Travel) | 2 | 2III_D.1.1 |
| OQ821378 | 2019 | 9 | 20 | 1 | 0 | Florida (USA) | Miami-Dade | 1 | 1III_A.3 |
| OQ821430 | 2019 | 9 | 21 | 0 | 1 | Cuba | Caribbean (Travel) | 2 | 2III_C.2 |
| OQ821431 | 2019 | 9 | 23 | 0 | 1 | Cuba | Caribbean (Travel) | 2 | 2III_C.2 |
| OQ821432 | 2019 | 9 | 23 | 0 | 1 | Cuba | Caribbean (Travel) | 2 | 2III_C.2 |
| OQ821433 | 2019 | 9 | 24 | 0 | 1 | Cuba | Caribbean (Travel) | 2 | 2III_C.2 |
| OQ821434 | 2019 | 9 | 26 | 0 | 1 | Cuba | Caribbean (Travel) | 2 | 2III_D.1.1 |
| OQ821435 | 2019 | 9 | 28 | 0 | 1 | Cuba | Caribbean (Travel) | 2 | 2III_D.1.1 |

medRxiv preprint doi: https://doi.org/10.64898/2026.05.01.26352185; this version posted May 5, 2026. The copyright holder for this preprint (which was not certified by peer review) is the author/funder, who has granted medRxiv a license to display the preprint in perpetuity.
It is made available under a CC-BY 4.0 International license .

| | | | | | | | | | |
|---|---|---|---|---|---|---|---|---|---|
| OQ821436 | 2019 | 9 | 29 | 0 | 1 | Cuba | Caribbean (Travel) | 2 | 2III_C.2 |
| OQ821437 | 2019 | 9 | 29 | 0 | 1 | Cuba | Caribbean (Travel) | 2 | 2III_C.2 |
| OQ821336 | 2019 | 10 | 2 | 0 | 1 | Cuba | Caribbean (Travel) | 1 | 1V_D.1.1 |
| OQ821337 | 2019 | 10 | 4 | 0 | 1 | Cuba | Caribbean (Travel) | 1 | 1V_D.1.1 |
| OQ821358 | 2019 | 10 | 8 | 0 | 1 | Dominican-Republic | Caribbean (Travel) | 1 | 1V_E.2 |
| OQ821438 | 2019 | 10 | 10 | 0 | 1 | Cuba | Caribbean (Travel) | 2 | 2III_C.2 |
| OQ821439 | 2019 | 10 | 13 | 0 | 1 | Cuba | Caribbean (Travel) | 2 | 2III_C.2 |
| OQ821440 | 2019 | 10 | 14 | 0 | 1 | Cuba | Caribbean (Travel) | 2 | 2III_C.2 |
| OQ821441 | 2019 | 10 | 14 | 0 | 1 | Cuba | Caribbean (Travel) | 2 | 2III_D.1.1 |
| OQ821359 | 2019 | 10 | 17 | 0 | 1 | Dominican-Republic | Caribbean (Travel) | 1 | 1V_E.2 |
| OQ821608 | 2019 | 10 | 19 | 0 | 1 | Jamaica | Caribbean (Travel) | 3 | 3III_B.3.1 |
| OQ821380 | 2019 | 10 | 23 | 0 | 1 | Venezuela | South-America (Travel) | 1 | 1V_D.1 |
| OQ821442 | 2019 | 10 | 28 | 0 | 1 | Cuba | Caribbean (Travel) | 2 | 2III_D.1.1 |
| OQ821609 | 2019 | 11 | 6 | 0 | 1 | Jamaica | Caribbean (Travel) | 3 | 3III_B.3.1 |
| OQ821484 | 2019 | 11 | 6 | 0 | 1 | Mexico | North-America (Travel) | 2 | 2III_D.1.2 |
| OQ821443 | 2019 | 11 | 9 | 0 | 1 | Cuba | Caribbean (Travel) | 2 | 2III_C.2 |
| OQ821444 | 2019 | 11 | 13 | 0 | 1 | Cuba | Caribbean (Travel) | 2 | 2III_C.2 |
| OQ821445 | 2019 | 11 | 13 | 0 | 1 | Cuba | Caribbean (Travel) | 2 | 2III_C.2 |
| OQ821446 | 2019 | 11 | 24 | 0 | 1 | Cuba | Caribbean (Travel) | 2 | 2III_C.2 |
| OQ821447 | 2019 | 11 | 25 | 0 | 1 | Cuba | Caribbean (Travel) | 2 | 2III_C.2 |
| OQ821366 | 2019 | 12 | 5 | 0 | 1 | Mexico | North-America (Travel) | 1 | 1V_D.1 |
| OQ821499 | 2019 | 12 | 10 | 1 | 0 | Florida (USA) | Miami-Dade | 2 | 2III_C.2 |
| OQ821448 | 2019 | 12 | 11 | 0 | 1 | Cuba | Caribbean (Travel) | 2 | 2III_C.2 |
| OQ821492 | 2019 | 12 | 13 | 0 | 1 | Nicaragua | Central-America (Travel) | 2 | 2III_D.1.2 |
| OQ821325 | 2020 | 1 | 9 | 0 | 1 | Bolivia | South-America (Travel) | 1 | 1V_D.2 |
| OQ821338 | 2020 | 1 | 10 | 0 | 1 | Cuba | Caribbean (Travel) | 1 | 1V_D.1.1 |
| OQ821449 | 2020 | 1 | 15 | 0 | 1 | Cuba | Caribbean (Travel) | 2 | 2III_C.2 |
| OQ821610 | 2020 | 1 | 17 | 0 | 1 | Jamaica | Caribbean (Travel) | 3 | 3III_B.3.1 |
| OQ821326 | 2020 | 3 | 15 | 0 | 1 | Bolivia | South-America (Travel) | 1 | 1V_D.2 |
| OQ821624 | 2020 | 3 | 19 | 0 | 1 | Paraguay | South-America (Travel) | 4 | 4II_B.1.1 |
| OM833056 | 2020 | 6 | 23 | 1 | 0 | Florida (USA) | Monroe | 1 | 1V_E.2 |
| OM833058 | 2020 | 6 | 27 | 1 | 0 | Florida (USA) | Monroe | 1 | 1V_E.2 |
| OM833057 | 2020 | 7 | 29 | 1 | 0 | Florida (USA) | Monroe | 1 | 1V_E.2 |
| OQ821611 | 2020 | 11 | 7 | 0 | 1 | Saint-Lucia | Caribbean (Travel) | 3 | 3III_B.3.1 |
| OQ821339 | 2020 | 11 | 25 | 0 | 1 | Cuba | Caribbean (Travel) | 1 | 1V_D.1.1 |
| OQ821365 | 2020 | 12 | 18 | 0 | 1 | Cuba | Caribbean (Travel) | 1 | 1V_D.1.1 |
| OQ821502 | 2021 | 8 | 12 | 0 | 1 | Colombia | South-America (Travel) | 3 | 3III_C.1 |
| OQ821340 | 2021 | 11 | 16 | 0 | 1 | Cuba | Caribbean (Travel) | 1 | 1V_E.2 |
| OQ821450 | 2021 | 12 | 22 | 0 | 1 | Cuba | Caribbean (Travel) | 2 | 2III_D.1.1 |
| OQ821451 | 2021 | 12 | 24 | 0 | 1 | Cuba | Caribbean (Travel) | 2 | 2III_D.1.1 |
| OQ821452 | 2022 | 1 | 30 | 0 | 1 | Cuba | Caribbean (Travel) | 2 | 2III_C.2 |
| OQ821453 | 2022 | 2 | 19 | 0 | 1 | Cuba | Caribbean (Travel) | 2 | 2III_D.1.1 |
| OQ821341 | 2022 | 4 | 24 | 0 | 1 | Cuba | Caribbean (Travel) | 1 | 1V_D.1.1 |

medRxiv preprint doi: https://doi.org/10.64898/2026.05.01.26352185; this version posted May 5, 2026. The copyright holder for this preprint (which was not certified by peer review) is the author/funder, who has granted medRxiv a license to display the preprint in perpetuity.
It is made available under a CC-BY 4.0 International license .

| | | | | | | | | | |
|---|---|---|---|---|---|---|---|---|---|
| OQ821342 | 2022 | 5 | 16 | 0 | 1 | Cuba | Caribbean (Travel) | 1 | 1V_D.1.1 |
| OQ821454 | 2022 | 6 | 13 | 0 | 1 | Cuba | Caribbean (Travel) | 2 | 2III_C.2 |
| OQ821455 | 2022 | 6 | 15 | 0 | 1 | Cuba | Caribbean (Travel) | 2 | 2III_C.2 |
| OQ821639 | 2022 | 6 | 15 | 0 | 1 | Cuba | Caribbean (Travel) | 4 | 4II_B.1.2 |
| OQ821343 | 2022 | 6 | 17 | 0 | 1 | Cuba | Caribbean (Travel) | 1 | 1V_D.1 |
| OQ821637 | 2022 | 6 | 19 | 0 | 1 | Cuba | Caribbean (Travel) | 4 | 4II_B.1.2 |
| OQ821456 | 2022 | 6 | 20 | 0 | 1 | Cuba | Caribbean (Travel) | 2 | 2III_D.1.1 |
| OQ821344 | 2022 | 6 | 23 | 0 | 1 | Cuba | Caribbean (Travel) | 1 | 1V_D.1.1 |
| OQ821642 | 2022 | 6 | 24 | 0 | 1 | Cuba | Caribbean (Travel) | 4 | 4II_B.1.2 |
| OQ821457 | 2022 | 6 | 28 | 0 | 1 | Cuba | Caribbean (Travel) | 2 | 2III_D.1.1 |
| OQ821458 | 2022 | 6 | 28 | 0 | 1 | Cuba | Caribbean (Travel) | 2 | 2III_D.1.1 |
| OQ821459 | 2022 | 6 | 28 | 0 | 1 | Cuba | Caribbean (Travel) | 2 | 2III_D.1.1 |
| OQ821329 | 2022 | 6 | 29 | 0 | 1 | Brazil | South-America (Travel) | 1 | 1V_E.1 |
| OQ821481 | 2022 | 7 | 1 | 0 | 1 | India | South-Asia (Travel) | 2 | 2II_F.1.1 |
| OQ821345 | 2022 | 7 | 2 | 0 | 1 | Cuba | Caribbean (Travel) | 1 | 1V_E.2 |
| OQ821460 | 2022 | 7 | 4 | 0 | 1 | Cuba | Caribbean (Travel) | 2 | 2III_C.2 |
| OQ821640 | 2022 | 7 | 5 | 0 | 1 | Cuba | Caribbean (Travel) | 4 | 4II_B.1.2 |
| OQ821461 | 2022 | 7 | 6 | 0 | 1 | Cuba | Caribbean (Travel) | 2 | 2III_C.2 |
| OQ821462 | 2022 | 7 | 6 | 0 | 1 | Cuba | Caribbean (Travel) | 2 | 2III_C.2 |
| OQ821504 | 2022 | 7 | 6 | 0 | 1 | Cuba | Caribbean (Travel) | 3 | 3III_B.3.2 |
| OQ821485 | 2022 | 7 | 6 | 0 | 1 | Mexico | North-America (Travel) | 2 | 2III_D.1.2 |
| OQ821641 | 2022 | 7 | 11 | 0 | 1 | Cuba | Caribbean (Travel) | 4 | 4II_B.1.2 |
| OQ445953 | 2022 | 7 | 13 | 1 | 0 | Florida (USA) | Miami-Dade | 3 | 3III_B.3.2 |
| OQ821346 | 2022 | 7 | 14 | 0 | 1 | Cuba | Caribbean (Travel) | 1 | 1V_D.1 |
| OQ821463 | 2022 | 7 | 14 | 0 | 1 | Cuba | Caribbean (Travel) | 2 | 2III_D.1.1 |
| OQ821643 | 2022 | 7 | 15 | 0 | 1 | Cuba | Caribbean (Travel) | 4 | 4II_B.1.2 |
| OQ821505 | 2022 | 7 | 17 | 0 | 1 | Cuba | Caribbean (Travel) | 3 | 3III_B.3.2 |
| OQ821638 | 2022 | 7 | 21 | 0 | 1 | Cuba | Caribbean (Travel) | 4 | 4II_B.1.2 |
| OQ821506 | 2022 | 7 | 23 | 0 | 1 | Cuba | Caribbean (Travel) | 3 | 3III_B.3.2 |
| OQ821464 | 2022 | 7 | 26 | 0 | 1 | Cuba | Caribbean (Travel) | 2 | 2III_D.1.1 |
| OQ821507 | 2022 | 7 | 28 | 0 | 1 | Cuba | Caribbean (Travel) | 3 | 3III_B.3.2 |
| OQ821508 | 2022 | 7 | 28 | 0 | 1 | Cuba | Caribbean (Travel) | 3 | 3III_B.3.2 |
| OQ445948 | 2022 | 7 | 29 | 1 | 0 | Florida (USA) | Miami-Dade | 3 | 3III_B.3.2 |
| OQ821347 | 2022 | 8 | 2 | 0 | 1 | Cuba | Caribbean (Travel) | 1 | 1V_D.1 |
| OQ821466 | 2022 | 8 | 5 | 0 | 1 | Cuba | Caribbean (Travel) | 2 | 2III_D.1.1 |
| OQ821509 | 2022 | 8 | 5 | 0 | 1 | Cuba | Caribbean (Travel) | 3 | 3III_B.3.2 |
| OQ445947 | 2022 | 8 | 5 | 1 | 0 | Florida (USA) | Miami-Dade | 3 | 3III_B.3.2 |
| OQ821510 | 2022 | 8 | 6 | 0 | 1 | Cuba | Caribbean (Travel) | 3 | 3III_B.3.2 |
| OQ821467 | 2022 | 8 | 8 | 0 | 1 | Cuba | Caribbean (Travel) | 2 | 2III_C.2 |
| OQ821613 | 2022 | 8 | 8 | 1 | 0 | Florida (USA) | Miami-Dade | 3 | 3III_B.3.2 |
| OQ821511 | 2022 | 8 | 9 | 0 | 1 | Cuba | Caribbean (Travel) | 3 | 3III_B.3.2 |
| OQ445918 | 2022 | 8 | 9 | 1 | 0 | Florida (USA) | Miami-Dade | 3 | 3III_B.3.2 |
| OQ445946 | 2022 | 8 | 9 | 1 | 0 | Florida (USA) | Miami-Dade | 3 | 3III_B.3.2 |

medRxiv preprint doi: https://doi.org/10.64898/2026.05.01.26352185; this version posted May 5, 2026. The copyright holder for this preprint (which was not certified by peer review) is the author/funder, who has granted medRxiv a license to display the preprint in perpetuity.
It is made available under a CC-BY 4.0 International license .

| | | | | | | | | | |
|---|---|---|---|---|---|---|---|---|---|
| OQ821512 | 2022 | 8 | 10 | 0 | 1 | Cuba | Caribbean (Travel) | 3 | 3III_B.3.2 |
| OQ821614 | 2022 | 8 | 11 | 1 | 0 | Florida (USA) | Miami-Dade | 3 | 3III_B.3.2 |
| OQ821513 | 2022 | 8 | 12 | 0 | 1 | Cuba | Caribbean (Travel) | 3 | 3III_B.3.2 |
| OQ821514 | 2022 | 8 | 13 | 0 | 1 | Cuba | Caribbean (Travel) | 3 | 3III_B.3.2 |
| OQ821515 | 2022 | 8 | 14 | 0 | 1 | Cuba | Caribbean (Travel) | 3 | 3III_B.3.2 |
| OQ821516 | 2022 | 8 | 15 | 0 | 1 | Cuba | Caribbean (Travel) | 3 | 3III_B.3.2 |
| OQ445919 | 2022 | 8 | 15 | 1 | 0 | Florida (USA) | Miami-Dade | 3 | 3III_B.3.2 |
| OQ821517 | 2022 | 8 | 16 | 0 | 1 | Cuba | Caribbean (Travel) | 3 | 3III_B.3.2 |
| OQ821615 | 2022 | 8 | 17 | 1 | 0 | Florida (USA) | Miami-Dade | 3 | 3III_B.3.2 |
| OQ821468 | 2022 | 8 | 18 | 0 | 1 | Cuba | Caribbean (Travel) | 2 | 2III_C.2 |
| OQ821518 | 2022 | 8 | 18 | 0 | 1 | Cuba | Caribbean (Travel) | 3 | 3III_B.3.2 |
| OQ821519 | 2022 | 8 | 18 | 0 | 1 | Cuba | Caribbean (Travel) | 3 | 3III_B.3.2 |
| OQ821520 | 2022 | 8 | 18 | 0 | 1 | Cuba | Caribbean (Travel) | 3 | 3III_B.3.2 |
| OQ821521 | 2022 | 8 | 18 | 0 | 1 | Cuba | Caribbean (Travel) | 3 | 3III_B.3.2 |
| OQ821522 | 2022 | 8 | 18 | 0 | 1 | Cuba | Caribbean (Travel) | 3 | 3III_B.3.2 |
| OQ821523 | 2022 | 8 | 18 | 0 | 1 | Cuba | Caribbean (Travel) | 3 | 3III_B.3.2 |
| OQ445920 | 2022 | 8 | 18 | 1 | 0 | Florida (USA) | Miami-Dade | 3 | 3III_B.3.2 |
| OQ821482 | 2022 | 8 | 19 | 0 | 1 | India | South-Asia (Travel) | 2 | 2II_F.1.1 |
| OQ821524 | 2022 | 8 | 20 | 0 | 1 | Cuba | Caribbean (Travel) | 3 | 3III_B.3.2 |
| OQ919693 | 2022 | 8 | 20 | 0 | 1 | Cuba | Caribbean (Travel) | 3 | 3III_B.3.2 |
| OQ821525 | 2022 | 8 | 21 | 0 | 1 | Cuba | Caribbean (Travel) | 3 | 3III_B.3.2 |
| OQ821526 | 2022 | 8 | 23 | 0 | 1 | Cuba | Caribbean (Travel) | 3 | 3III_B.3.2 |
| OQ821527 | 2022 | 8 | 23 | 0 | 1 | Cuba | Caribbean (Travel) | 3 | 3III_B.3.2 |
| OQ821528 | 2022 | 8 | 24 | 0 | 1 | Cuba | Caribbean (Travel) | 3 | 3III_B.3.2 |
| OQ821529 | 2022 | 8 | 24 | 0 | 1 | Cuba | Caribbean (Travel) | 3 | 3III_B.3.2 |
| OQ821530 | 2022 | 8 | 24 | 0 | 1 | Cuba | Caribbean (Travel) | 3 | 3III_B.3.2 |
| OQ821646 | 2022 | 8 | 24 | 0 | 1 | El-Salvador | Central-America (Travel) | 4 | 4II_B.2 |
| OQ821469 | 2022 | 8 | 26 | 0 | 1 | Cuba | Caribbean (Travel) | 2 | 2III_C.2 |
| OQ821348 | 2022 | 8 | 28 | 0 | 1 | Cuba | Caribbean (Travel) | 1 | 1V_D.1.1 |
| OQ821531 | 2022 | 8 | 28 | 0 | 1 | Cuba | Caribbean (Travel) | 3 | 3III_B.3.2 |
| OQ445921 | 2022 | 8 | 28 | 1 | 0 | Florida (USA) | Miami-Dade | 3 | 3III_B.3.2 |
| OQ445922 | 2022 | 8 | 29 | 1 | 0 | Florida (USA) | Miami-Dade | 3 | 3III_B.3.2 |
| OQ821532 | 2022 | 8 | 30 | 0 | 1 | Cuba | Caribbean (Travel) | 3 | 3III_B.3.2 |
| OQ821533 | 2022 | 8 | 31 | 0 | 1 | Cuba | Caribbean (Travel) | 3 | 3III_B.3.2 |
| OQ821534 | 2022 | 8 | 31 | 0 | 1 | Cuba | Caribbean (Travel) | 3 | 3III_B.3.2 |
| OQ821349 | 2022 | 9 | 1 | 0 | 1 | Cuba | Caribbean (Travel) | 1 | 1V_D.1.1 |
| OQ821535 | 2022 | 9 | 1 | 0 | 1 | Cuba | Caribbean (Travel) | 3 | 3III_B.3.2 |
| OQ821536 | 2022 | 9 | 3 | 0 | 1 | Cuba | Caribbean (Travel) | 3 | 3III_B.3.2 |
| OQ821473 | 2022 | 9 | 3 | 0 | 1 | Dominican-Republic | Caribbean (Travel) | 2 | 2III_C.1.2 |
| OQ821537 | 2022 | 9 | 5 | 0 | 1 | Cuba | Caribbean (Travel) | 3 | 3III_B.3.2 |
| OQ821538 | 2022 | 9 | 5 | 0 | 1 | Cuba | Caribbean (Travel) | 3 | 3III_B.3.2 |
| OQ445923 | 2022 | 9 | 5 | 1 | 0 | Florida (USA) | Miami-Dade | 3 | 3III_B.3.2 |
| OQ821539 | 2022 | 9 | 7 | 0 | 1 | Cuba | Caribbean (Travel) | 3 | 3III_B.3.2 |

medRxiv preprint doi: https://doi.org/10.64898/2026.05.01.26352185; this version posted May 5, 2026. The copyright holder for this preprint (which was not certified by peer review) is the author/funder, who has granted medRxiv a license to display the preprint in perpetuity.
It is made available under a CC-BY 4.0 International license .

| | | | | | | | | | |
|---|---|---|---|---|---|---|---|---|---|
| OQ821540 | 2022 | 9 | 8 | 0 | 1 | Cuba | Caribbean (Travel) | 3 | 3III_B.3.2 |
| OQ821645 | 2022 | 9 | 10 | 0 | 1 | Nicaragua | Central-America (Travel) | 4 | 4II_B.2 |
| OQ821541 | 2022 | 9 | 12 | 0 | 1 | Cuba | Caribbean (Travel) | 3 | 3III_B.3.2 |
| OQ821542 | 2022 | 9 | 13 | 0 | 1 | Cuba | Caribbean (Travel) | 3 | 3III_B.3.2 |
| OQ821543 | 2022 | 9 | 13 | 0 | 1 | Cuba | Caribbean (Travel) | 3 | 3III_B.3.2 |
| OQ821544 | 2022 | 9 | 14 | 0 | 1 | Cuba | Caribbean (Travel) | 3 | 3III_B.3.2 |
| OQ445925 | 2022 | 9 | 14 | 1 | 0 | Florida (USA) | Miami-Dade | 3 | 3III_B.3.2 |
| OQ821545 | 2022 | 9 | 15 | 0 | 1 | Cuba | Caribbean (Travel) | 3 | 3III_B.3.2 |
| OQ821546 | 2022 | 9 | 17 | 0 | 1 | Cuba | Caribbean (Travel) | 3 | 3III_B.3.2 |
| OQ821547 | 2022 | 9 | 17 | 0 | 1 | Cuba | Caribbean (Travel) | 3 | 3III_B.3.2 |
| OQ821350 | 2022 | 9 | 19 | 0 | 1 | Cuba | Caribbean (Travel) | 1 | 1V_D.1 |
| OQ821548 | 2022 | 9 | 21 | 0 | 1 | Cuba | Caribbean (Travel) | 3 | 3III_B.3.2 |
| OQ821549 | 2022 | 9 | 22 | 0 | 1 | Cuba | Caribbean (Travel) | 3 | 3III_B.3.2 |
| OQ821550 | 2022 | 9 | 22 | 0 | 1 | Cuba | Caribbean (Travel) | 3 | 3III_B.3.2 |
| OQ919689 | 2022 | 9 | 22 | 1 | 0 | Florida (USA) | Miami-Dade | 3 | 3III_B.3.2 |
| OQ821551 | 2022 | 9 | 23 | 0 | 1 | Cuba | Caribbean (Travel) | 3 | 3III_B.3.2 |
| OQ821552 | 2022 | 9 | 23 | 0 | 1 | Cuba | Caribbean (Travel) | 3 | 3III_B.3.2 |
| OQ821553 | 2022 | 9 | 23 | 0 | 1 | Cuba | Caribbean (Travel) | 3 | 3III_B.3.2 |
| OQ821554 | 2022 | 9 | 25 | 0 | 1 | Cuba | Caribbean (Travel) | 3 | 3III_B.3.2 |
| OQ821555 | 2022 | 9 | 25 | 0 | 1 | Cuba | Caribbean (Travel) | 3 | 3III_B.3.2 |
| OQ821556 | 2022 | 9 | 25 | 0 | 1 | Cuba | Caribbean (Travel) | 3 | 3III_B.3.2 |
| OQ445927 | 2022 | 9 | 25 | 1 | 0 | Florida (USA) | Miami-Dade | 3 | 3III_B.3.2 |
| OQ445929 | 2022 | 9 | 25 | 1 | 0 | Florida (USA) | Miami-Dade | 3 | 3III_B.3.2 |
| OQ821470 | 2022 | 9 | 26 | 0 | 1 | Cuba | Caribbean (Travel) | 2 | 2III_D.1.1 |
| OQ821557 | 2022 | 9 | 26 | 0 | 1 | Cuba | Caribbean (Travel) | 3 | 3III_B.3.2 |
| OQ821558 | 2022 | 9 | 26 | 0 | 1 | Cuba | Caribbean (Travel) | 3 | 3III_B.3.2 |
| OR150743 | 2022 | 9 | 26 | 1 | 0 | Florida (USA) | Miami-Dade | 2 | 2III_D.1.1 |
| OQ821559 | 2022 | 9 | 29 | 0 | 1 | Cuba | Caribbean (Travel) | 3 | 3III_B.3.2 |
| OQ821560 | 2022 | 9 | 29 | 0 | 1 | Cuba | Caribbean (Travel) | 3 | 3III_B.3.2 |
| OQ821561 | 2022 | 9 | 30 | 0 | 1 | Cuba | Caribbean (Travel) | 3 | 3III_B.3.2 |
| OQ821562 | 2022 | 10 | 2 | 0 | 1 | Cuba | Caribbean (Travel) | 3 | 3III_B.3.2 |
| OQ821616 | 2022 | 10 | 4 | 1 | 0 | Florida (USA) | Miami-Dade | 3 | 3III_B.3.2 |
| OQ821563 | 2022 | 10 | 5 | 0 | 1 | Cuba | Caribbean (Travel) | 3 | 3III_B.3.2 |
| OQ919690 | 2022 | 10 | 5 | 0 | 1 | Cuba | Caribbean (Travel) | 3 | 3III_B.3.2 |
| OQ821564 | 2022 | 10 | 7 | 0 | 1 | Cuba | Caribbean (Travel) | 3 | 3III_B.3.2 |
| OQ445930 | 2022 | 10 | 10 | 1 | 0 | Florida (USA) | Miami-Dade | 3 | 3III_B.3.2 |
| OQ821617 | 2022 | 10 | 10 | 1 | 0 | Florida (USA) | Miami-Dade | 3 | 3III_B.3.2 |
| OQ821565 | 2022 | 10 | 14 | 0 | 1 | Cuba | Caribbean (Travel) | 3 | 3III_B.3.2 |
| OQ821566 | 2022 | 10 | 15 | 0 | 1 | Cuba | Caribbean (Travel) | 3 | 3III_B.3.2 |
| OQ821618 | 2022 | 10 | 15 | 1 | 0 | Florida (USA) | Miami-Dade | 3 | 3III_B.3.2 |
| OQ821567 | 2022 | 10 | 19 | 0 | 1 | Cuba | Caribbean (Travel) | 3 | 3III_B.3.2 |
| OQ445931 | 2022 | 10 | 19 | 1 | 0 | Florida (USA) | Miami-Dade | 3 | 3III_B.3.2 |
| OQ821568 | 2022 | 10 | 20 | 0 | 1 | Cuba | Caribbean (Travel) | 3 | 3III_B.3.2 |

medRxiv preprint doi: https://doi.org/10.64898/2026.05.01.26352185; this version posted May 5, 2026. The copyright holder for this preprint (which was not certified by peer review) is the author/funder, who has granted medRxiv a license to display the preprint in perpetuity.
It is made available under a CC-BY 4.0 International license .

| | | | | | | | | | |
|---|---|---|---|---|---|---|---|---|---|
| OQ445934 | 2022 | 10 | 20 | 1 | 0 | Florida (USA) | Miami-Dade | 3 | 3III_B.3.2 |
| OQ821569 | 2022 | 10 | 21 | 0 | 1 | Cuba | Caribbean (Travel) | 3 | 3III_B.3.2 |
| OQ821644 | 2022 | 10 | 22 | 0 | 1 | Nicaragua | Central-America (Travel) | 4 | 4II_B.2 |
| OQ445932 | 2022 | 10 | 23 | 1 | 0 | Florida (USA) | Miami-Dade | 3 | 3III_B.3.2 |
| OQ445933 | 2022 | 10 | 24 | 1 | 0 | Florida (USA) | Miami-Dade | 3 | 3III_B.3.2 |
| OQ445935 | 2022 | 10 | 26 | 1 | 0 | Florida (USA) | Miami-Dade | 3 | 3III_B.3.2 |
| OQ821570 | 2022 | 10 | 30 | 0 | 1 | Cuba | Caribbean (Travel) | 3 | 3III_B.3.2 |
| OQ821571 | 2022 | 11 | 3 | 0 | 1 | Cuba | Caribbean (Travel) | 3 | 3III_B.3.2 |
| OQ821572 | 2022 | 11 | 3 | 0 | 1 | Cuba | Caribbean (Travel) | 3 | 3III_B.3.2 |
| OQ445967 | 2022 | 11 | 3 | 1 | 0 | Florida (USA) | Miami-Dade | 4 | 4II_B.2 |
| OQ821573 | 2022 | 11 | 4 | 0 | 1 | Cuba | Caribbean (Travel) | 3 | 3III_B.3.2 |
| OQ821574 | 2022 | 11 | 7 | 0 | 1 | Cuba | Caribbean (Travel) | 3 | 3III_B.3.2 |
| OQ919691 | 2022 | 11 | 8 | 1 | 0 | Florida (USA) | Miami-Dade | 3 | 3III_B.3.2 |
| OQ821575 | 2022 | 11 | 10 | 0 | 1 | Cuba | Caribbean (Travel) | 3 | 3III_B.3.2 |
| OQ821576 | 2022 | 11 | 14 | 0 | 1 | Cuba | Caribbean (Travel) | 3 | 3III_B.3.2 |
| OQ821636 | 2022 | 11 | 15 | 0 | 1 | Cuba | Caribbean (Travel) | 4 | 4II_B.1.2 |
| OR162320 | 2022 | 11 | 15 | 1 | 0 | Florida (USA) | Miami-Dade | 4 | 4II_B.1.2 |
| OQ821619 | 2022 | 11 | 17 | 1 | 0 | Florida (USA) | Miami-Dade | 3 | 3III_B.3.2 |
| OQ821577 | 2022 | 11 | 18 | 0 | 1 | Cuba | Caribbean (Travel) | 3 | 3III_B.3.2 |
| OQ821578 | 2022 | 11 | 18 | 0 | 1 | Cuba | Caribbean (Travel) | 3 | 3III_B.3.2 |
| OQ821579 | 2022 | 11 | 20 | 0 | 1 | Cuba | Caribbean (Travel) | 3 | 3III_B.3.2 |
| OQ821620 | 2022 | 11 | 21 | 1 | 0 | Florida (USA) | Miami-Dade | 3 | 3III_B.3.2 |
| OQ821580 | 2022 | 11 | 25 | 0 | 1 | Cuba | Caribbean (Travel) | 3 | 3III_B.3.2 |
| PP709312 | 2022 | 11 | 25 | 1 | 0 | Florida (USA) | Miami-Dade | 3 | 3III_B.3.2 |
| OR162311 | 2022 | 11 | 27 | 1 | 0 | Florida (USA) | Miami-Dade | 3 | 3III_B.3.2 |
| OR162313 | 2022 | 11 | 27 | 1 | 0 | Florida (USA) | Miami-Dade | 3 | 3III_B.3.2 |
| OQ821621 | 2022 | 11 | 28 | 1 | 0 | Florida (USA) | Miami-Dade | 3 | 3III_B.3.2 |
| OQ821581 | 2022 | 11 | 29 | 0 | 1 | Cuba | Caribbean (Travel) | 3 | 3III_B.3.2 |
| OQ821471 | 2022 | 11 | 30 | 0 | 1 | Cuba | Caribbean (Travel) | 2 | 2III_D.1.1 |
| OQ821582 | 2022 | 11 | 30 | 0 | 1 | Cuba | Caribbean (Travel) | 3 | 3III_B.3.2 |
| OQ821583 | 2022 | 11 | 30 | 0 | 1 | Cuba | Caribbean (Travel) | 3 | 3III_B.3.2 |
| OQ821584 | 2022 | 11 | 30 | 0 | 1 | Cuba | Caribbean (Travel) | 3 | 3III_B.3.2 |
| OQ821585 | 2022 | 12 | 2 | 0 | 1 | Cuba | Caribbean (Travel) | 3 | 3III_B.3.2 |
| OR162312 | 2022 | 12 | 2 | 1 | 0 | Florida (USA) | Miami-Dade | 3 | 3III_B.3.2 |
| OR162316 | 2022 | 12 | 2 | 1 | 0 | Florida (USA) | Miami-Dade | 3 | 3III_B.3.2 |
| OQ821351 | 2022 | 12 | 3 | 0 | 1 | Cuba | Caribbean (Travel) | 1 | 1V_D.1.1 |
| OQ821586 | 2022 | 12 | 4 | 0 | 1 | Cuba | Caribbean (Travel) | 3 | 3III_B.3.2 |
| OQ821587 | 2022 | 12 | 4 | 0 | 1 | Cuba | Caribbean (Travel) | 3 | 3III_B.3.2 |
| OQ919692 | 2022 | 12 | 4 | 0 | 1 | Cuba | Caribbean (Travel) | 3 | 3III_B.3.2 |
| OR162314 | 2022 | 12 | 4 | 1 | 0 | Florida (USA) | Miami-Dade | 3 | 3III_B.3.2 |
| OR162317 | 2022 | 12 | 4 | 1 | 0 | Florida (USA) | Miami-Dade | 3 | 3III_B.3.2 |
| OQ821588 | 2022 | 12 | 6 | 0 | 1 | Cuba | Caribbean (Travel) | 3 | 3III_B.3.2 |
| OQ821589 | 2022 | 12 | 6 | 0 | 1 | Cuba | Caribbean (Travel) | 3 | 3III_B.3.2 |

medRxiv preprint doi: https://doi.org/10.64898/2026.05.01.26352185; this version posted May 5, 2026. The copyright holder for this preprint (which was not certified by peer review) is the author/funder, who has granted medRxiv a license to display the preprint in perpetuity.
It is made available under a CC-BY 4.0 International license .

| | | | | | | | | | |
|---|---|---|---|---|---|---|---|---|---|
| OR162315 | 2022 | 12 | 6 | 1 | 0 | Florida (USA) | Miami-Dade | 3 | 3III_B.3.2 |
| OQ821352 | 2022 | 12 | 7 | 0 | 1 | Cuba | Caribbean (Travel) | 1 | 1V_D.1.1 |
| OQ821367 | 2022 | 12 | 7 | 0 | 1 | Mexico | North-America (Travel) | 1 | 1V_D.1.2 |
| OQ821590 | 2022 | 12 | 8 | 0 | 1 | Cuba | Caribbean (Travel) | 3 | 3III_B.3.2 |
| OR162318 | 2022 | 12 | 10 | 1 | 0 | Florida (USA) | Miami-Dade | 3 | 3III_B.3.2 |
| OQ821622 | 2022 | 12 | 11 | 1 | 0 | Florida (USA) | Miami-Dade | 3 | 3III_B.3.2 |
| OQ821591 | 2022 | 12 | 15 | 0 | 1 | Cuba | Caribbean (Travel) | 3 | 3III_B.3.2 |
| OQ821592 | 2022 | 12 | 16 | 0 | 1 | Cuba | Caribbean (Travel) | 3 | 3III_B.3.2 |
| OQ821593 | 2022 | 12 | 16 | 0 | 1 | Cuba | Caribbean (Travel) | 3 | 3III_B.3.2 |
| OQ821594 | 2022 | 12 | 18 | 0 | 1 | Cuba | Caribbean (Travel) | 3 | 3III_B.3.2 |
| OQ821595 | 2022 | 12 | 18 | 0 | 1 | Cuba | Caribbean (Travel) | 3 | 3III_B.3.2 |
| OQ821596 | 2022 | 12 | 18 | 0 | 1 | Cuba | Caribbean (Travel) | 3 | 3III_B.3.2 |
| OR150746 | 2022 | 12 | 18 | 1 | 0 | Florida (USA) | Miami-Dade | 3 | 3III_B.3.2 |
| OR162319 | 2022 | 12 | 18 | 1 | 0 | Florida (USA) | Miami-Dade | 3 | 3III_B.3.2 |
| OR150744 | 2022 | 12 | 19 | 1 | 0 | Florida (USA) | Miami-Dade | 3 | 3III_B.3.2 |
| OQ821635 | 2022 | 12 | 20 | 0 | 1 | Cuba | Caribbean (Travel) | 4 | 4II_B.1.2 |
| OQ821597 | 2022 | 12 | 21 | 0 | 1 | Cuba | Caribbean (Travel) | 3 | 3III_B.3.2 |
| OQ821598 | 2022 | 12 | 22 | 0 | 1 | Cuba | Caribbean (Travel) | 3 | 3III_B.3.2 |
| OQ821599 | 2022 | 12 | 22 | 0 | 1 | Cuba | Caribbean (Travel) | 3 | 3III_B.3.2 |
| OR150745 | 2022 | 12 | 22 | 1 | 0 | Florida (USA) | Miami-Dade | 3 | 3III_B.3.2 |
| OQ821623 | 2022 | 12 | 23 | 1 | 0 | Florida (USA) | Miami-Dade | 3 | 3III_B.3.2 |
| OQ821600 | 2023 | 1 | 1 | 0 | 1 | Cuba | Caribbean (Travel) | 3 | 3III_B.3.2 |
| OR150748 | 2023 | 1 | 1 | 1 | 0 | Florida (USA) | Polk | 3 | 3III_B.3.2 |
| OR150749 | 2023 | 1 | 6 | 1 | 0 | Florida (USA) | Orange | 3 | 3III_B.3.2 |
| OR654284 | 2023 | 7 | 3 | 1 | 0 | Florida (USA) | Miami-Dade | 2 | 2II_F.1.1.2 |
| OR771126 | 2023 | 7 | 12 | 1 | 0 | Florida (USA) | Miami-Dade | 3 | 3III_B.3.2 |
| OR771131 | 2023 | 7 | 26 | 1 | 0 | Florida (USA) | Miami-Dade | 3 | 3III_B.3.2 |
| OR771136 | 2023 | 7 | 27 | 1 | 0 | Florida (USA) | Miami-Dade | 3 | 3III_B.3.2 |
| OR771144 | 2023 | 8 | 4 | 1 | 0 | Florida (USA) | Miami-Dade | 3 | 3III_B.3.2 |
| OR771158 | 2023 | 8 | 22 | 1 | 0 | Florida (USA) | Miami-Dade | 3 | 3III_B.3.2 |
| OR771167 | 2023 | 8 | 31 | 1 | 0 | Florida (USA) | Miami-Dade | 3 | 3III_B.3.2 |
| OR771177 | 2023 | 9 | 6 | 1 | 0 | Florida (USA) | Miami-Dade | 3 | 3III_B.3.2 |
| OR771179 | 2023 | 9 | 11 | 1 | 0 | Florida (USA) | Miami-Dade | 3 | 3III_B.3.2 |
| OR771180 | 2023 | 9 | 11 | 1 | 0 | Florida (USA) | Miami-Dade | 3 | 3III_B.3.2 |
| OR771183 | 2023 | 9 | 12 | 1 | 0 | Florida (USA) | Miami-Dade | 2 | 2II_F.1.1.2 |
| OR771181 | 2023 | 9 | 12 | 1 | 0 | Florida (USA) | Miami-Dade | 3 | 3III_B.3.2 |
| OR771185 | 2023 | 9 | 16 | 1 | 0 | Florida (USA) | Miami-Dade | 3 | 3III_B.3.2 |
| OR821940 | 2023 | 9 | 19 | 1 | 0 | Florida (USA) | Miami-Dade | 3 | 3III_B.3.2 |
| OR771182 | 2023 | 9 | 20 | 1 | 0 | Florida (USA) | Miami-Dade | 3 | 3III_B.3.2 |
| OR821936 | 2023 | 9 | 21 | 1 | 0 | Florida (USA) | Miami-Dade | 3 | 3III_B.3.2 |
| OR771188 | 2023 | 9 | 26 | 1 | 0 | Florida (USA) | Miami-Dade | 2 | 2II_F.1.1.2 |
| OR771189 | 2023 | 9 | 26 | 1 | 0 | Florida (USA) | Miami-Dade | 2 | 2II_F.1.1 |
| OR821944 | 2023 | 9 | 26 | 1 | 0 | Florida (USA) | Miami-Dade | 2 | 2II_F.1.1.2 |

medRxiv preprint doi: https://doi.org/10.64898/2026.05.01.26352185; this version posted May 5, 2026. The copyright holder for this preprint (which was not certified by peer review) is the author/funder, who has granted medRxiv a license to display the preprint in perpetuity.
It is made available under a CC-BY 4.0 International license .

| | | | | | | | | | |
|---|---|---|---|---|---|---|---|---|---|
| OR771195 | 2023 | 9 | 26 | 1 | 0 | Florida (USA) | Miami-Dade | 3 | 3III_B.3.2 |
| OR821941 | 2023 | 9 | 26 | 1 | 0 | Florida (USA) | Miami-Dade | 3 | 3III_B.3.2 |
| OR821942 | 2023 | 9 | 27 | 1 | 0 | Florida (USA) | Miami-Dade | 3 | 3III_B.3.2 |
| OR821946 | 2023 | 9 | 28 | 1 | 0 | Florida (USA) | Miami-Dade | 2 | 2II_F.1.1.2 |
| OR821945 | 2023 | 9 | 28 | 1 | 0 | Florida (USA) | Miami-Dade | 3 | 3III_B.3.2 |
| OR771190 | 2023 | 9 | 29 | 1 | 0 | Florida (USA) | Miami-Dade | 3 | 3III_B.3.2 |
| OR771193 | 2023 | 9 | 30 | 1 | 0 | Florida (USA) | Miami-Dade | 2 | 2II_F.1.1 |
| OR771191 | 2023 | 9 | 30 | 1 | 0 | Florida (USA) | Miami-Dade | 3 | 3III_B.3.2 |
| OR821948 | 2023 | 9 | 30 | 1 | 0 | Florida (USA) | Miami-Dade | 3 | 3III_B.3.2 |
| OR821950 | 2023 | 10 | 2 | 1 | 0 | Florida (USA) | Miami-Dade | 3 | 3III_B.3.2 |
| OR771199 | 2023 | 10 | 3 | 1 | 0 | Florida (USA) | Miami-Dade | 3 | 3III_B.3.2 |
| OR771192 | 2023 | 10 | 4 | 1 | 0 | Florida (USA) | Miami-Dade | 3 | 3III_B.3.2 |
| OR821952 | 2023 | 10 | 5 | 1 | 0 | Florida (USA) | Miami-Dade | 3 | 3III_B.3.2 |
| OR821949 | 2023 | 10 | 6 | 1 | 0 | Florida (USA) | Miami-Dade | 3 | 3III_B.3.2 |
| OR821951 | 2023 | 10 | 7 | 1 | 0 | Florida (USA) | Miami-Dade | 3 | 3III_B.3.2 |
| OR821955 | 2023 | 10 | 9 | 1 | 0 | Florida (USA) | Miami-Dade | 3 | 3III_B.3.2 |
| OR821954 | 2023 | 10 | 10 | 1 | 0 | Florida (USA) | Miami-Dade | 3 | 3III_B.3.2 |
| OR821958 | 2023 | 10 | 10 | 1 | 0 | Florida (USA) | Miami-Dade | 3 | 3III_B.3.2 |
| OR821962 | 2023 | 10 | 11 | 1 | 0 | Florida (USA) | Miami-Dade | 2 | 2II_F.1.1.5 |
| OR977072 | 2023 | 10 | 13 | 1 | 0 | Florida (USA) | Miami-Dade | 3 | 3III_B.3.2 |
| OR821964 | 2023 | 10 | 14 | 1 | 0 | Florida (USA) | Miami-Dade | 3 | 3III_B.3.2 |
| OR977076 | 2023 | 10 | 17 | 1 | 0 | Florida (USA) | Miami-Dade | 3 | 3III_B.3.2 |
| OR977080 | 2023 | 10 | 20 | 1 | 0 | Florida (USA) | Miami-Dade | 2 | 2II_F.1.1 |
| OR977077 | 2023 | 10 | 22 | 1 | 0 | Florida (USA) | Miami-Dade | 3 | 3III_B.3.2 |
| OR977081 | 2023 | 10 | 25 | 1 | 0 | Florida (USA) | Miami-Dade | 3 | 3III_B.3.2 |
| OR977085 | 2023 | 10 | 28 | 1 | 0 | Florida (USA) | Miami-Dade | 3 | 3III_B.3.2 |
| OR977086 | 2023 | 11 | 1 | 1 | 0 | Florida (USA) | Miami-Dade | 3 | 3III_B.3.2 |
| OR977088 | 2023 | 11 | 7 | 1 | 0 | Florida (USA) | Miami-Dade | 3 | 3III_B.3.2 |
| PP692455 | 2023 | 11 | 12 | 1 | 0 | Florida (USA) | Miami-Dade | 3 | 3III_B.3.2 |
| OR977091 | 2023 | 11 | 15 | 1 | 0 | Florida (USA) | Miami-Dade | 3 | 3III_B.3.2 |
| OR977092 | 2023 | 11 | 15 | 1 | 0 | Florida (USA) | Miami-Dade | 3 | 3III_B.3.2 |
| OR977099 | 2023 | 11 | 19 | 1 | 0 | Florida (USA) | Miami-Dade | 3 | 3III_B.3.2 |
| PP709309 | 2023 | 11 | 20 | 1 | 0 | Florida (USA) | Hardee | 1 | 1V_D.1 |
| OR977097 | 2023 | 11 | 20 | 1 | 0 | Florida (USA) | Miami-Dade | 3 | 3III_B.3.2 |
| PP692465 | 2023 | 12 | 12 | 1 | 0 | Florida (USA) | Miami-Dade | 3 | 3III_B.3.2 |
| PP708029 | 2024 | 1 | 2 | 1 | 0 | Florida (USA) | Miami-Dade | 3 | 3III_B.3.2 |
| PP708030 | 2024 | 1 | 8 | 1 | 0 | Florida (USA) | Miami-Dade | 3 | 3III_B.3.2 |
| PP708038 | 2024 | 1 | 20 | 1 | 0 | Florida (USA) | Miami-Dade | 3 | 3III_B.3.2 |
| PQ340822 | 2024 | 6 | 25 | 1 | 0 | Florida (USA) | Miami-Dade | 3 | 3III_B.3.2 |
| PQ527045 | 2024 | 8 | 13 | 1 | 0 | Florida (USA) | Hillsborough | 3 | 3III_B.3.2 |
| PQ617129 | 2024 | 8 | 26 | 1 | 0 | Florida (USA) | Pasco | 3 | 3III_B.3.2 |
| PQ617146 | 2024 | 9 | 8 | 1 | 0 | Florida (USA) | Miami-Dade | 3 | 3III_B.3.2 |
| PQ617120 | 2024 | 9 | 25 | 1 | 0 | Florida (USA) | Palm-Beach | 3 | 3III_B.3.2 |

medRxiv preprint doi: https://doi.org/10.64898/2026.05.01.26352185; this version posted May 5, 2026. The copyright holder for this preprint
(which was not certified by peer review) is the author/funder, who has granted medRxiv a license to display the preprint in perpetuity.
It is made available under a CC-BY 4.0 International license .

medRxiv preprint doi: https://doi.org/10.64898/2026.05.01.26352185; this version posted May 5, 2026. The copyright holder for this preprint (which was not certified by peer review) is the author/funder, who has granted medRxiv a license to display the preprint in perpetuity.
It is made available under a CC-BY 4.0 International license .

**Table S3. Transmission clusters of local DENV cases across counties in Florida**. This includes information on the county in which the cases were reported (sample region), major lineage, cluster size, inferred introduction sources and time, and the earliest and latest sampling dates of local dengue cases.

| Transmission cluster | Sample region (DTA) | Major lineage | Cluster size | Source region (DTA) | Posterior probability of source region | Median introduction time | Introduction time 95% credible interval | Earliest local sample date | Latest local sample date |
|---|---|---|---|---|---|---|---|---|---|
| Cluster 1 | Monroe | 1V_C | 8 | Central-America | 1 | 2010.1 | (2009.35,2010.44) | 2010.48 | 2010.88 |
| Cluster 2 | Miami-Dade | 2II_F | 4 | South-America | 1 | 2023.48 | (2023.28,2023.62) | 2023.7 | 2023.74 |
| Cluster 3 | Miami-Dade | 3III_B | 4 | Caribbean | 0.85 | 2021.95 | (2021.71,2022.13) | 2022.65 | 2022.81 |
| Cluster 4 | Miami-Dade | 3III_B | 27 | Caribbean | 1 | 2023.27 | (2023.09,2023.45) | 2023.64 | 2024.05 |
| Cluster 5 | Miami-Dade | 3III_B | 12 | Caribbean | 0.94 | 2023.31 | (2023.09,2023.54) | 2023.69 | 2024.02 |
| Cluster 6 | Miami-Dade | 3III_B | 6 | Caribbean | 0.72 | 2022.57 | (2022.37,2022.71) | 2022.72 | 2022.91 |

medRxiv preprint doi: https://doi.org/10.64898/2026.05.01.26352185; this version posted May 5, 2026. The copyright holder for this preprint (which was not certified by peer review) is the author/funder, who has granted medRxiv a license to display the preprint in perpetuity.
It is made available under a CC-BY 4.0 International license .

**Table S4 Genomic sequences sampled from locally-acquired dengue infections in Florida from 2010 to 2024 and their inferred introduction sources and times from DTA analysis.** This includes their GenBank accession numbers, sample collection date, assigned major lineage, trait used in DTA analysis (sample region (DTA)). The following columns are populated from our DTA analysis, including their inferred trait (source region (DTA)), posterior probability of inferred trait, transmission cluster membership, and introduction time (median height of transition node and its corresponding 95% credible interval). Introduction times can only be inferred for when a transition node has two or more descending tips belonging to a local dengue sample. Transmission cluster membership is defined by transition nodes with three or more descending tips belonging to a local dengue sample.

| GenBank Accession | Collection date | Collection date (decimal date) | Major lineage | Sample region (DTA) | Source region (DTA) | Posterior probability of source region | Transmission cluster | Median introduction time | Introduction time 95% credible interval |
|---|---|---|---|---|---|---|---|---|---|
| OQ821370 | 6/24/10 | 2010.48 | 1V_C | Monroe | Central-America | 1 | Cluster 1 | 2010.1 | (2009.35,2010.44) |
| OQ821371 | 6/30/10 | 2010.49 | 1V_C | Monroe | Central-America | 1 | Cluster 1 | 2010.1 | (2009.35,2010.44) |
| OQ821372 | 8/6/10 | 2010.59 | 1V_C | Monroe | Central-America | 1 | Cluster 1 | 2010.1 | (2009.35,2010.44) |
| OQ821373 | 8/11/10 | 2010.61 | 1V_C | Monroe | Central-America | 1 | Cluster 1 | 2010.1 | (2009.35,2010.44) |
| OQ821374 | 8/27/10 | 2010.65 | 1V_C | Monroe | Central-America | 1 | Cluster 1 | 2010.1 | (2009.35,2010.44) |
| JQ675358 | 2010-10 | 2010.75 | 1V_C | Monroe | Central-America | 1 | Cluster 1 | 2010.1 | (2009.35,2010.44) |
| OQ821375 | 10/11/10 | 2010.78 | 1V_C | Monroe | Central-America | 1 | Cluster 1 | 2010.1 | (2009.35,2010.44) |
| OQ821376 | 11/20/10 | 2010.88 | 1V_C | Monroe | Central-America | 1 | Cluster 1 | 2010.1 | (2009.35,2010.44) |
| OM833059 | 7/24/13 | 2013.56 | 1V_E | Monroe | Caribbean | 1 | | | |
| OQ821377 | 8/29/13 | 2013.66 | 1V_E | Martin | Caribbean | 0.9 | | | |
| OQ821612 | 7/2/14 | 2014.5 | 3I_A | Miami-Dade | Global | 1 | | | |
| MN192436 | 2016 | 2016 | 4II_B | Manatee | Caribbean | 0.99 | | | |
| KX702404 | 5/22/16 | 2016.39 | 2III_C | Alachua | South-America | 1 | | | |
| OQ821496 | 7/11/19 | 2019.52 | 2III_D | Miami-Dade | Caribbean | 1 | | | |
| OQ821497 | 8/29/19 | 2019.66 | 2III_D | Miami-Dade | Central-America | 1 | | | |
| OQ821498 | 9/14/19 | 2019.7 | 2III_D | Miami-Dade | Central-America | 0.97 | | | |
| OQ821378 | 9/20/19 | 2019.72 | 1III_A | Miami-Dade | Global | 1 | | | |
| OQ821499 | 12/10/19 | 2019.94 | 2III_C | Miami-Dade | Caribbean | 1 | | | |
| OM833056 | 6/23/20 | 2020.48 | 1V_E | Monroe | Caribbean | 0.98 | | 2020.04 | (2019.25,2020.36) |
| OM833058 | 6/27/20 | 2020.49 | 1V_E | Monroe | Caribbean | 0.95 | | | |
| OM833057 | 7/29/20 | 2020.57 | 1V_E | Monroe | Caribbean | 0.98 | | 2020.04 | (2019.25,2020.36) |
| OQ445953 | 7/13/22 | 2022.53 | 3III_B | Miami-Dade | Caribbean | 1 | | | |
| OQ445948 | 7/29/22 | 2022.57 | 3III_B | Miami-Dade | Caribbean | 1 | | 2022.31 | (2021.99,2022.51) |
| OQ445947 | 8/5/22 | 2022.59 | 3III_B | Miami-Dade | Caribbean | 1 | | | |
| OQ445918 | 8/9/22 | 2022.6 | 3III_B | Miami-Dade | Caribbean | 0.96 | | 2021.86 | (2021.29,2022.25) |
| OQ445946 | 8/9/22 | 2022.6 | 3III_B | Miami-Dade | Caribbean | 1 | | 2022.31 | (2021.99,2022.51) |
| OQ821613 | 8/8/22 | 2022.6 | 3III_B | Miami-Dade | Caribbean | 0.99 | | | |
| OQ821614 | 8/11/22 | 2022.61 | 3III_B | Miami-Dade | Caribbean | 0.88 | | | |

medRxiv preprint doi: https://doi.org/10.64898/2026.05.01.26352185; this version posted May 5, 2026. The copyright holder for this preprint (which was not certified by peer review) is the author/funder, who has granted medRxiv a license to display the preprint in perpetuity.
It is made available under a CC-BY 4.0 International license .

| | | | | | | | | | |
|---|---|---|---|---|---|---|---|---|---|
| OQ445919 | 8/15/22 | 2022.62 | 3III_B | Miami-Dade | Caribbean | 0.96 | | 2021.86 | (2021.29,2022.25) |
| OQ821615 | 8/17/22 | 2022.62 | 3III_B | Miami-Dade | Caribbean | 1 | | | |
| OQ445920 | 8/18/22 | 2022.63 | 3III_B | Miami-Dade | Caribbean | 0.93 | | | |
| OQ445921 | 8/28/22 | 2022.65 | 3III_B | Miami-Dade | Caribbean | 0.85 | Cluster 3 | 2021.95 | (2021.71,2022.13) |
| OQ445922 | 8/29/22 | 2022.66 | 3III_B | Miami-Dade | Caribbean | 0.94 | | | |
| OQ445923 | 9/5/22 | 2022.68 | 3III_B | Miami-Dade | Caribbean | 0.95 | | | |
| OQ445925 | 9/14/22 | 2022.7 | 3III_B | Miami-Dade | Caribbean | 1 | | | |
| OQ919689 | 9/22/22 | 2022.72 | 3III_B | Miami-Dade | Caribbean | 0.72 | Cluster 6 | 2022.57 | (2022.37,2022.71) |
| OR150743 | 9/26/22 | 2022.73 | 2III_D | Miami-Dade | Caribbean | 1 | | | |
| OQ445927 | 9/25/22 | 2022.73 | 3III_B | Miami-Dade | Caribbean | 0.85 | | | |
| OQ445929 | 9/25/22 | 2022.73 | 3III_B | Miami-Dade | Caribbean | 0.89 | | | |
| OQ821616 | 10/4/22 | 2022.76 | 3III_B | Miami-Dade | Caribbean | 0.72 | Cluster 6 | 2022.57 | (2022.37,2022.71) |
| OQ445930 | 10/10/22 | 2022.77 | 3III_B | Miami-Dade | Caribbean | 0.85 | Cluster 3 | 2021.95 | (2021.71,2022.13) |
| OQ821617 | 10/10/22 | 2022.77 | 3III_B | Miami-Dade | Caribbean | 1 | | | |
| OQ821618 | 10/15/22 | 2022.79 | 3III_B | Miami-Dade | Caribbean | 0.72 | Cluster 6 | 2022.57 | (2022.37,2022.71) |
| OQ445931 | 10/19/22 | 2022.8 | 3III_B | Miami-Dade | Caribbean | 0.85 | Cluster 3 | 2021.95 | (2021.71,2022.13) |
| OQ445934 | 10/20/22 | 2022.8 | 3III_B | Miami-Dade | Caribbean | 0.93 | | 2022.2 | (2021.89,2022.57) |
| OQ445932 | 10/23/22 | 2022.81 | 3III_B | Miami-Dade | Caribbean | 0.95 | | | |
| OQ445933 | 10/24/22 | 2022.81 | 3III_B | Miami-Dade | Caribbean | 0.85 | Cluster 3 | 2021.95 | (2021.71,2022.13) |
| OQ445935 | 10/26/22 | 2022.82 | 3III_B | Miami-Dade | Caribbean | 0.93 | | 2022.2 | (2021.89,2022.57) |
| OQ445967 | 11/3/22 | 2022.84 | 4II_B | Miami-Dade | North-America | 0.82 | | | |
| OQ919691 | 11/8/22 | 2022.85 | 3III_B | Miami-Dade | Caribbean | 1 | | 2022.81 | (2022.65,2022.85) |
| OR162320 | 11/15/22 | 2022.87 | 4II_B | Miami-Dade | Caribbean | 0.67 | | | |
| OQ821619 | 11/17/22 | 2022.88 | 3III_B | Miami-Dade | Caribbean | 0.72 | Cluster 6 | 2022.57 | (2022.37,2022.71) |
| OQ821620 | 11/21/22 | 2022.89 | 3III_B | Miami-Dade | Caribbean | 0.72 | Cluster 6 | 2022.57 | (2022.37,2022.71) |
| OR162311 | 11/27/22 | 2022.9 | 3III_B | Miami-Dade | Caribbean | 0.73 | | | |
| OR162313 | 11/27/22 | 2022.9 | 3III_B | Miami-Dade | Caribbean | 1 | | 2022.71 | (2022.37,2022.89) |
| PP709312 | 11/25/22 | 2022.9 | 3III_B | Miami-Dade | Caribbean | 0.7 | | | |
| OQ821621 | 11/28/22 | 2022.91 | 3III_B | Miami-Dade | Caribbean | 0.72 | Cluster 6 | 2022.57 | (2022.37,2022.71) |
| OR162312 | 12/2/22 | 2022.92 | 3III_B | Miami-Dade | Caribbean | 0.6 | | | |
| OR162314 | 12/4/22 | 2022.92 | 3III_B | Miami-Dade | Caribbean | 1 | | | |
| OR162316 | 12/2/22 | 2022.92 | 3III_B | Miami-Dade | Caribbean | 1 | | | |
| OR162317 | 12/4/22 | 2022.92 | 3III_B | Miami-Dade | Caribbean | 1 | | | |
| OR162315 | 12/6/22 | 2022.93 | 3III_B | Miami-Dade | Caribbean | 1 | | | |
| OQ821622 | 12/11/22 | 2022.94 | 3III_B | Miami-Dade | Caribbean | 1 | | 2022.81 | (2022.65,2022.85) |
| OR162318 | 12/10/22 | 2022.94 | 3III_B | Miami-Dade | Caribbean | 1 | | 2022.71 | (2022.37,2022.89) |
| OR150744 | 12/19/22 | 2022.96 | 3III_B | Miami-Dade | Caribbean | 1 | | | |
| OR150746 | 12/18/22 | 2022.96 | 3III_B | Miami-Dade | Caribbean | 1 | | | |
| OR162319 | 12/18/22 | 2022.96 | 3III_B | Miami-Dade | Caribbean | 1 | | | |
| OR150745 | 12/22/22 | 2022.97 | 3III_B | Miami-Dade | Caribbean | 1 | | | |

medRxiv preprint doi: https://doi.org/10.64898/2026.05.01.26352185; this version posted May 5, 2026. The copyright holder for this preprint (which was not certified by peer review) is the author/funder, who has granted medRxiv a license to display the preprint in perpetuity.
It is made available under a CC-BY 4.0 International license .

| | | | | | | | | | |
|---|---|---|---|---|---|---|---|---|---|
| OQ821623 | 12/23/22 | 2022.98 | 3III_B | Miami-Dade | Caribbean | 1 | | | |
| OR150748 | 1/1/23 | 2023 | 3III_B | Polk | Caribbean | 1 | | | |
| OR150749 | 1/6/23 | 2023.01 | 3III_B | Orange | Caribbean | 0.99 | | | |
| OR654284 | 7/3/23 | 2023.5 | 2II_F | Miami-Dade | South-America | 1 | | | |
| OR771126 | 7/12/23 | 2023.53 | 3III_B | Miami-Dade | Caribbean | 0.89 | | | |
| OR771131 | 7/26/23 | 2023.56 | 3III_B | Miami-Dade | Caribbean | 0.94 | | | |
| OR771136 | 7/27/23 | 2023.57 | 3III_B | Miami-Dade | Caribbean | 1 | | | |
| OR771144 | 8/4/23 | 2023.59 | 3III_B | Miami-Dade | Caribbean | 1 | | | |
| OR771158 | 8/22/23 | 2023.64 | 3III_B | Miami-Dade | Caribbean | 1 | Cluster 4 | 2023.27 | (2023.09,2023.45) |
| OR771167 | 8/31/23 | 2023.66 | 3III_B | Miami-Dade | Caribbean | 1 | Cluster 4 | 2023.27 | (2023.09,2023.45) |
| OR771177 | 9/6/23 | 2023.68 | 3III_B | Miami-Dade | Caribbean | 1 | Cluster 4 | 2023.27 | (2023.09,2023.45) |
| OR771179 | 9/11/23 | 2023.69 | 3III_B | Miami-Dade | Caribbean | 0.94 | Cluster 5 | 2023.31 | (2023.09,2023.54) |
| OR771180 | 9/11/23 | 2023.69 | 3III_B | Miami-Dade | Caribbean | 1 | Cluster 4 | 2023.27 | (2023.09,2023.45) |
| OR771183 | 9/12/23 | 2023.7 | 2II_F | Miami-Dade | South-America | 1 | Cluster 2 | 2023.48 | (2023.28,2023.62) |
| OR771181 | 9/12/23 | 2023.7 | 3III_B | Miami-Dade | Caribbean | 0.94 | Cluster 5 | 2023.31 | (2023.09,2023.54) |
| OR771185 | 9/16/23 | 2023.71 | 3III_B | Miami-Dade | Caribbean | 0.99 | | 2023.63 | (2023.47,2023.7) |
| OR771182 | 9/20/23 | 2023.72 | 3III_B | Miami-Dade | Caribbean | 1 | Cluster 4 | 2023.27 | (2023.09,2023.45) |
| OR821936 | 9/21/23 | 2023.72 | 3III_B | Miami-Dade | Caribbean | 0.99 | | 2023.63 | (2023.47,2023.7) |
| OR821940 | 9/19/23 | 2023.72 | 3III_B | Miami-Dade | Caribbean | 1 | Cluster 4 | 2023.27 | (2023.09,2023.45) |
| OR771188 | 9/26/23 | 2023.73 | 2II_F | Miami-Dade | South-America | 1 | Cluster 2 | 2023.48 | (2023.28,2023.62) |
| OR771189 | 9/26/23 | 2023.73 | 2II_F | Miami-Dade | Caribbean | 0.95 | | | |
| OR821944 | 9/26/23 | 2023.73 | 2II_F | Miami-Dade | South-America | 1 | Cluster 2 | 2023.48 | (2023.28,2023.62) |
| OR771195 | 9/26/23 | 2023.73 | 3III_B | Miami-Dade | Caribbean | 1 | Cluster 4 | 2023.27 | (2023.09,2023.45) |
| OR821941 | 9/26/23 | 2023.73 | 3III_B | Miami-Dade | Caribbean | 1 | Cluster 4 | 2023.27 | (2023.09,2023.45) |
| OR821946 | 9/28/23 | 2023.74 | 2II_F | Miami-Dade | South-America | 1 | Cluster 2 | 2023.48 | (2023.28,2023.62) |
| OR771190 | 9/29/23 | 2023.74 | 3III_B | Miami-Dade | Caribbean | 1 | Cluster 4 | 2023.27 | (2023.09,2023.45) |
| OR821942 | 9/27/23 | 2023.74 | 3III_B | Miami-Dade | Caribbean | 1 | Cluster 4 | 2023.27 | (2023.09,2023.45) |
| OR821945 | 9/28/23 | 2023.74 | 3III_B | Miami-Dade | Caribbean | 1 | Cluster 4 | 2023.27 | (2023.09,2023.45) |
| OR771193 | 9/30/23 | 2023.75 | 2II_F | Miami-Dade | Caribbean | 0.95 | | | |
| OR771191 | 9/30/23 | 2023.75 | 3III_B | Miami-Dade | Caribbean | 1 | Cluster 4 | 2023.27 | (2023.09,2023.45) |
| OR771199 | 10/3/23 | 2023.75 | 3III_B | Miami-Dade | Caribbean | 1 | Cluster 4 | 2023.27 | (2023.09,2023.45) |
| OR821948 | 9/30/23 | 2023.75 | 3III_B | Miami-Dade | Caribbean | 1 | Cluster 4 | 2023.27 | (2023.09,2023.45) |
| OR821950 | 10/2/23 | 2023.75 | 3III_B | Miami-Dade | Caribbean | 1 | Cluster 4 | 2023.27 | (2023.09,2023.45) |
| OR771192 | 10/4/23 | 2023.76 | 3III_B | Miami-Dade | Caribbean | 0.94 | Cluster 5 | 2023.31 | (2023.09,2023.54) |
| OR821949 | 10/6/23 | 2023.76 | 3III_B | Miami-Dade | Caribbean | 1 | Cluster 4 | 2023.27 | (2023.09,2023.45) |
| OR821951 | 10/7/23 | 2023.76 | 3III_B | Miami-Dade | Caribbean | 1 | Cluster 4 | 2023.27 | (2023.09,2023.45) |
| OR821952 | 10/5/23 | 2023.76 | 3III_B | Miami-Dade | Caribbean | 1 | Cluster 4 | 2023.27 | (2023.09,2023.45) |
| OR821954 | 10/10/23 | 2023.77 | 3III_B | Miami-Dade | Caribbean | 0.94 | Cluster 5 | 2023.31 | (2023.09,2023.54) |
| OR821955 | 10/9/23 | 2023.77 | 3III_B | Miami-Dade | Caribbean | 1 | Cluster 4 | 2023.27 | (2023.09,2023.45) |
| OR821958 | 10/10/23 | 2023.77 | 3III_B | Miami-Dade | Caribbean | 1 | Cluster 4 | 2023.27 | (2023.09,2023.45) |

medRxiv preprint doi: https://doi.org/10.64898/2026.05.01.26352185; this version posted May 5, 2026. The copyright holder for this preprint (which was not certified by peer review) is the author/funder, who has granted medRxiv a license to display the preprint in perpetuity.
It is made available under a CC-BY 4.0 International license .

| | | | | | | | | | |
|---|---|---|---|---|---|---|---|---|---|
| OR821962 | 10/11/23 | 2023.78 | 2II_F | Miami-Dade | Caribbean | 1 | | | |
| OR821964 | 10/14/23 | 2023.78 | 3III_B | Miami-Dade | Caribbean | 1 | Cluster 4 | 2023.27 | (2023.09,2023.45) |
| OR977072 | 10/13/23 | 2023.78 | 3III_B | Miami-Dade | Caribbean | 1 | Cluster 4 | 2023.27 | (2023.09,2023.45) |
| OR977076 | 10/17/23 | 2023.79 | 3III_B | Miami-Dade | Caribbean | 1 | Cluster 4 | 2023.27 | (2023.09,2023.45) |
| OR977080 | 10/20/23 | 2023.8 | 2II_F | Miami-Dade | Caribbean | 1 | | | |
| OR977077 | 10/22/23 | 2023.81 | 3III_B | Miami-Dade | Caribbean | 1 | Cluster 4 | 2023.27 | (2023.09,2023.45) |
| OR977081 | 10/25/23 | 2023.81 | 3III_B | Miami-Dade | Caribbean | 0.94 | Cluster 5 | 2023.31 | (2023.09,2023.54) |
| OR977085 | 10/28/23 | 2023.82 | 3III_B | Miami-Dade | Caribbean | 0.94 | Cluster 5 | 2023.31 | (2023.09,2023.54) |
| OR977086 | 11/1/23 | 2023.83 | 3III_B | Miami-Dade | Caribbean | 1 | Cluster 4 | 2023.27 | (2023.09,2023.45) |
| OR977088 | 11/7/23 | 2023.85 | 3III_B | Miami-Dade | Caribbean | 0.94 | Cluster 5 | 2023.31 | (2023.09,2023.54) |
| PP692455 | 11/12/23 | 2023.86 | 3III_B | Miami-Dade | Caribbean | 0.94 | Cluster 5 | 2023.31 | (2023.09,2023.54) |
| OR977091 | 11/15/23 | 2023.87 | 3III_B | Miami-Dade | Caribbean | 0.94 | Cluster 5 | 2023.31 | (2023.09,2023.54) |
| OR977092 | 11/15/23 | 2023.87 | 3III_B | Miami-Dade | Caribbean | 0.94 | Cluster 5 | 2023.31 | (2023.09,2023.54) |
| PP709309 | 11/20/23 | 2023.88 | 1V_D | Hardee | North-America | 0.98 | | | |
| OR977097 | 11/20/23 | 2023.88 | 3III_B | Miami-Dade | Caribbean | 0.94 | Cluster 5 | 2023.31 | (2023.09,2023.54) |
| OR977099 | 11/19/23 | 2023.88 | 3III_B | Miami-Dade | Caribbean | 1 | Cluster 4 | 2023.27 | (2023.09,2023.45) |
| PP692465 | 12/12/23 | 2023.95 | 3III_B | Miami-Dade | Caribbean | 0.89 | | | |
| PP708029 | 1/2/24 | 2024 | 3III_B | Miami-Dade | Caribbean | 1 | | | |
| PP708030 | 1/8/24 | 2024.02 | 3III_B | Miami-Dade | Caribbean | 0.94 | Cluster 5 | 2023.31 | (2023.09,2023.54) |
| PP708038 | 1/20/24 | 2024.05 | 3III_B | Miami-Dade | Caribbean | 1 | Cluster 4 | 2023.27 | (2023.09,2023.45) |
| PQ340822 | 6/25/24 | 2024.48 | 3III_B | Miami-Dade | Caribbean | 1 | | | |
| PQ527045 | 8/13/24 | 2024.61 | 3III_B | Hillsborough | Caribbean | 1 | | | |
| PQ617129 | 8/26/24 | 2024.65 | 3III_B | Pasco | Caribbean | 1 | | | |
| PQ617146 | 9/8/24 | 2024.69 | 3III_B | Miami-Dade | Caribbean | 1 | | | |
| PQ617120 | 9/25/24 | 2024.73 | 3III_B | Palm-Beach | Caribbean | 0.71 | | | |

medRxiv preprint doi: https://doi.org/10.64898/2026.05.01.26352185; this version posted May 5, 2026. The copyright holder for this preprint (which was not certified by peer review) is the author/funder, who has granted medRxiv a license to display the preprint in perpetuity.
It is made available under a CC-BY 4.0 International license .

**Table S5 Covariates representing hypothesized drivers of local dengue infections in Florida.**

| Covariate | Model | Category | Source | Rationale |
|---|---|---|---|---|
| Annual mean, minimum, and maximum temperature | Occurrence | Climate | Visual Crossing (www.visualcrossing.com/) | Constrains geographical distribution of dengue virus and its mosquito vectors to known limits on temperature range for survival. Relationships may be non-linear. |
| Annual mean and total precipitation, number of days without precipitation, and annual mean relative humidity | Occurrence | Climate | Visual Crossing (www.visualcrossing.com/) | Moderate levels of precipitation and relative humidity may be optimal for dengue transmission, by acting on mosquito larval habitat availability. Lower levels may reduce habitat availability with fewer water bodies, and higher levels may increase risks of washing away habitats via flushing effects (*67*). |
| Annual travel-associated dengue cases per county | Occurrence | Travel cases | FDOH (*7*) | High travel volume between Florida and dengue-endemic regions like the Caribbean is a risk factor for local dengue cases (*8*) |
| Population density (county-level population estimates) | Occurrence | Population | U.S. Census Bureau (www.census.gov) | High population densities should correspond to greater contact rates and probabilities for long transmission chains. |
| Social vulnerability index (SVI) | Occurrence | SVI | CDC (*63*) | Summary statistics calculated from 16 demographic and socioeconomic census variables, where higher values reflect poverty, lack of accessible transportation, and more crowded housing. Rapid unplanned urbanization is a risk factor for dengue outbreaks, where high population densities often co-occur with weak water infrastructure and vector controls (*68*). |
| Annual mean and maximum index P, and number of days in a year with index P > 0 | Occurrence | Index P | Nakase *et al. (25)* | Proxy for DENV transmission suitability by adult female *Ae. aegypti*. Calculated using MVSE package (*25*). A greater number of days with index P > 0 reflects a longer period for suitable transmission. |
| Monthly mean, minimum, and maximum temperature | Incidence | Temperature | Visual Crossing (www.visualcrossing.com/) | Impacts temperature-dependent virus and mosquito traits for dengue transmission. Relationships are likely non-linear, and possible delays are tested from 0 to 6 months. |
| Monthly travel-associated dengue cases reported in Miami-Dade County | Incidence | Travel cases | FDOH (*7*) | High travel volume between Florida and dengue-endemic regions like the Caribbean is a risk factor for local dengue cases (*8*) |
| Monthly number of female *Ae. aegypti* per trap in Miami-Dade County | Incidence | Mosquito abundance | Miami-Dade County Mosquito Control Division | Higher abundance of adult female Ae. aegypti mosquitoes, competent vectors for dengue transmission, should increase risk of local dengue transmission. |
| Monthly mean and total precipitation, number of days without precipitation, and monthly mean relative humidity | Incidence | Hydrometeorologica l | Visual Crossing (www.visualcrossing.com/) | Moderate levels of precipitation and relative humidity may be optimal for dengue transmission, by acting on mosquito larval habitat availability. Lower levels may reduce habitat availability with fewer water bodies, and higher levels may increase risks of washing away habitats via flushing effects (*67*). |
| Monthly mean, minimum,and maximum index P, and number of days in a month with index P > 0 | Incidence | Index P | Nakase et al. 2023 (*25*) | Proxy for overall climate suitability for dengue transmission by adult female *Aedes. aegypti* mosquitoes, quantifies transmission potential from relative air humidity and temperature. Calculated in MVSE package (*25*). A greater number of days in a month with index P > 0 reflects a longer period of climate suitability for dengue transmission by *Ae. aegypti*. |

medRxiv preprint doi: https://doi.org/10.64898/2026.05.01.26352185; this version posted May 5, 2026. The copyright holder for this preprint (which was not certified by peer review) is the author/funder, who has granted medRxiv a license to display the preprint in perpetuity.
It is made available under a CC-BY 4.0 International license .

**Table S6. Default biological parameters used in estimating index P for DENV transmitted by *Ae. aegypti* mosquitoes from humidity (μ) and temperature (t)** (from Nakase *et al.*)

| **Parameter** | **Symbol** | **Distribution** | **Mean, SD** | **Units** |
|---|---|---|---|---|
| Adult *Ae. aegypti* lifespan | $1/\mu^{v}_{(u,t)}$ | normal | 10, 2.55 | days |
| Adult *Ae. aegypti* biting rate | $a^{v}_{(u)}$ | normal | 0.25, 0.01 | bites · mosq.$^{-1}$ · day$^{-1}$ |
| Human lifespan | $1/\mu^{h}$ | normal | 70, 3 | years |
| Intrinsic human-DENV incubation period | $1/\gamma^{h}$ | lognormal | 5.94, 1.80 | days |
| Human-DENV infectious period | $1/\sigma^{h}$ | normal | 4, 0.51 | days |
| Extrinsic *Ae. aegypti*-DENV incubation period | $1/\gamma^{v}_{(t)}$ | lognormal | 1228, 1825 (∞,15 °C)<br>232, 181 [15 °C, 17.5 °C)<br>72.4, 32.6 [17.5 °C, 20 °C)<br>28.9, 7.64 [20 °C, 22.5 °C)<br>14.7, 2.66 [22.5 °C, 25 °C)<br>8.68, 1.31 [25 °C, 27.5 °C)<br>5.76, 0.87 [27.5 °C, 30 °C)<br>4.14, 0.63 [30 °C, 32.5 °C)<br>3.19, 0.48 [32.5 °C, ∞) | days |

medRxiv preprint doi: https://doi.org/10.64898/2026.05.01.26352185; this version posted May 5, 2026. The copyright holder for this preprint (which was not certified by peer review) is the author/funder, who has granted medRxiv a license to display the preprint in perpetuity.
It is made available under a CC-BY 4.0 International license .

**Table S7. Summary metrics for baseline and univariate models of monthly local dengue case incidence in Miami-Dade County from 2009 to 2024, the latter fitted as a baseline model with the inclusion of a single covariate.** Models are ordered by ascending WAIC values, where larger values indicate a worse fit to the observed data**.** Covariates are categorized by type and functional form. Non-linear effects were modeled as a second-order random walk over a varying number of unique covariate values (5, 10, 20, and 40), where a greater number allowed for more flexibility.

| Covariate | Category | Form | Number of unique covariate values | WAIC difference from baseline |
|---|---|---|---|---|
| Baseline model | NA | NA | NA | 0 |
| Number of Ae. aegypti mosquitoes per trap | Mosquito abundance | Non-linear | 5 | -29.19 |
| Monthly travel case count | Travel cases | Non-linear | 40 | -27.04 |
| Monthly travel case count | Travel cases | Non-linear | 10 | -23.16 |
| Number of Ae. aegypti mosquitoes per trap | Mosquito abundance | Non-linear | 10 | -22.80 |
| Monthly travel case count | Travel cases | Non-linear | 20 | -20.72 |
| Monthly mean humidity | Hydrometeorological | Non-linear | 5 | -12.79 |
| Number of days with zero precipitation | Hydrometeorological | Non-linear | 40 | -10.31 |
| Monthly total precipitation | Hydrometeorological | Linear | NA | -9.51 |
| Monthly mean precipitation | Hydrometeorological | Linear | NA | -9.30 |
| Monthly mean index P | Index P | Non-linear | 5 | -8.83 |
| Monthly mean index P | Index P | Non-linear | 10 | -8.06 |
| Number of days with zero precipitation | Hydrometeorological | Non-linear | 20 | -6.72 |
| Number of Ae. aegypti mosquitoes per trap | Mosquito abundance | Non-linear | 20 | -6.49 |
| Number of days with zero precipitation | Hydrometeorological | Non-linear | 10 | -6.32 |
| Number of days with zero precipitation | Hydrometeorological | Linear | NA | -6.30 |
| Monthly travel case count | Travel cases | Log | NA | -6.29 |
| Monthly mean precipitation | Hydrometeorological | Non-linear | 40 | -5.88 |

medRxiv preprint doi: https://doi.org/10.64898/2026.05.01.26352185; this version posted May 5, 2026. The copyright holder for this preprint (which was not certified by peer review) is the author/funder, who has granted medRxiv a license to display the preprint in perpetuity.
It is made available under a CC-BY 4.0 International license .

| | | | | |
|---|---|---|---|---|
| Monthly maximum temperature | Temperature | Non-linear | 40 | -5.78 |
| Monthly total precipitation | Hydrometeorological | Non-linear | 40 | -5.71 |
| Monthly minimum temperature | Temperature | Non-linear | 20 | -5.10 |
| Monthly travel case count | Travel cases | Non-linear | 5 | -5.06 |
| Monthly mean precipitation | Hydrometeorological | Non-linear | 20 | -4.92 |
| Monthly total precipitation | Hydrometeorological | Non-linear | 5 | -4.91 |
| Monthly minimum temperature | Temperature | Non-linear | 10 | -4.87 |
| Monthly mean humidity | Hydrometeorological | Non-linear | 40 | -4.66 |
| Monthly mean humidity | Hydrometeorological | Non-linear | 10 | -4.52 |
| Monthly minimum temperature | Temperature | Non-linear | 5 | -4.50 |
| Monthly total precipitation | Hydrometeorological | Non-linear | 10 | -4.44 |
| Monthly total precipitation | Hydrometeorological | Non-linear | 20 | -4.42 |
| Monthly mean index P | Index P | Non-linear | 20 | -4.12 |
| Monthly mean precipitation | Hydrometeorological | Non-linear | 5 | -3.90 |
| Monthly mean humidity | Hydrometeorological | Non-linear | 20 | -3.77 |
| Monthly maximum temperature | Temperature | Non-linear | 20 | -3.59 |
| Monthly mean precipitation | Hydrometeorological | Non-linear | 10 | -3.35 |
| Monthly minimum index P | Index P | Linear | NA | -3.35 |
| Monthly mean index P | Index P | Non-linear | 40 | -2.33 |
| Number of days with zero precipitation | Hydrometeorological | Non-linear | 5 | -2.29 |
| Monthly mean humidity | Hydrometeorological | Linear | NA | -2.29 |
| Monthly maximum temperature | Temperature | Non-linear | 5 | -2.07 |
| Monthly minimum temperature | Temperature | Non-linear | 40 | -0.87 |
| Number of Ae. aegypti | Mosquito abundance | Non-linear | 40 | -0.62 |

medRxiv preprint doi: https://doi.org/10.64898/2026.05.01.26352185; this version posted May 5, 2026. The copyright holder for this preprint (which was not certified by peer review) is the author/funder, who has granted medRxiv a license to display the preprint in perpetuity.
It is made available under a CC-BY 4.0 International license .

| | | | | |
|---|---|---|---|---|
| mosquitoes per trap | | | | |
| Number of Ae. aegypti mosquitoes per trap | Mosquito abundance | Linear | NA | -0.41 |
| Monthly mean temperature | Temperature | Non-linear | 40 | -0.36 |
| Monthly number of days with index P > 0 | Index P | Non-linear | 40 | -0.22 |
| Monthly mean index P | Index P | Linear | NA | -0.20 |
| Monthly mean temperature | Temperature | Non-linear | 20 | -0.12 |
| Monthly maximum temperature | Temperature | Linear | NA | 0.04 |
| Monthly number of days with index P > 0 | Index P | Linear | NA | 0.14 |
| Monthly maximum temperature | Temperature | Non-linear | 10 | 0.19 |
| Monthly minimum temperature | Temperature | Linear | NA | 0.62 |
| Monthly number of days with index P > 0 | Index P | Non-linear | 20 | 0.69 |
| Monthly number of days with index P > 0 | Index P | Non-linear | 10 | 0.92 |
| Monthly mean temperature | Temperature | Non-linear | 10 | 1.23 |
| Monthly number of days with index P > 0 | Index P | Non-linear | 5 | 1.26 |
| Monthly maximum index P | Index P | Non-linear | 5 | 1.44 |
| Monthly mean temperature | Temperature | Non-linear | 5 | 2.09 |
| Monthly mean temperature | Temperature | Linear | NA | 2.75 |
| Monthly maximum index P | Index P | Non-linear | 10 | 2.79 |
| Monthly maximum index P | Index P | Linear | NA | 3.04 |
| Monthly maximum index P | Index P | Non-linear | 20 | 3.11 |
| Consecutive number of days with index P > 0 | Index P | Linear | NA | 3.14 |
| Consecutive number of days with index P > 0 | Index P | Non-linear | 40 | 3.19 |

medRxiv preprint doi: https://doi.org/10.64898/2026.05.01.26352185; this version posted May 5, 2026. The copyright holder for this preprint (which was not certified by peer review) is the author/funder, who has granted medRxiv a license to display the preprint in perpetuity.
It is made available under a CC-BY 4.0 International license .

| | | | | |
|---|---|---|---|---|
| Consecutive number of days with index P > 0 | Index P | Non-linear | 5 | 3.22 |
| Consecutive number of days with index P > 0 | Index P | Non-linear | 20 | 3.61 |
| Consecutive number of days with index P > 0 | Index P | Non-linear | 10 | 3.79 |
| Monthly maximum index P | Index P | Non-linear | 40 | 5.34 |
| Monthly travel case count | Travel cases | Linear | NA | 7.27 |
| Monthly minimum index P | Index P | Non-linear | 5 | 7.60 |
| Monthly minimum index P | Index P | Non-linear | 40 | 8.72 |
| Monthly minimum index P | Index P | Non-linear | 10 | 9.04 |
| Monthly minimum index P | Index P | Non-linear | 20 | 9.16 |
